# Renewable and tuneable bio-LPG blends derived from amino acids

**DOI:** 10.1186/s13068-020-01766-0

**Published:** 2020-07-14

**Authors:** Mohamed Amer, Robin Hoeven, Paul Kelly, Matthew Faulkner, Michael H. Smith, Helen S. Toogood, Nigel S. Scrutton

**Affiliations:** 1 EPSRC/BBSRC Future Biomanufacturing Research Hub, BBSRC/EPSRC, Synthetic Biology Research Centre SYNBIOCHEM Manchester Institute of Biotechnology and School of Chemistry, The University of Manchester, Manchester, M1 7DN UK; 2 C3 Biotechnologies Ltd, The Railway Goods Yard, Middleton-in-Lonsdale, Lancashire, LA6 2NF UK

**Keywords:** Biofuels, Propane, Isobutane, Butane, Microbial pathway engineering, *Escherichia coli*, Bio-LPG

## Abstract

**Background:**

Microbial biorefinery approaches are beginning to define renewable and sustainable routes to clean-burning and non-fossil fuel-derived gaseous alkanes (known as ‘bio-LPG’). The most promising strategies have used a terminal fatty acid photodecarboxylase, enabling light-driven propane production from externally fed waste butyric acid. Use of *Halomonas* (a robust extremophile microbial chassis) with these pathways has enabled bio-LPG production under non-sterile conditions and using waste biomass as the carbon source. Here, we describe new engineering approaches to produce next-generation pathways that use amino acids as fuel precursors for bio-LPG production (propane, butane and isobutane blends).

**Results:**

Multiple pathways from the amino acids valine, leucine and isoleucine were designed in *E. coli* for the production of propane, isobutane and butane, respectively. A branched-chain keto acid decarboxylase-dependent pathway utilising fatty acid photodecarboxylase was the most effective route, generating higher alkane gas titres over alternative routes requiring coenzyme A and/or aldehyde deformylating oxygenase. Isobutane was the major gas produced in standard (mixed amino acid) medium, however valine supplementation led to primarily propane production. Transitioning pathways into *Halomonas* strain TQ10 enabled fermentative production of mixed alkane gases under non-sterile conditions on simple carbon sources. Chromosomal integration of inducible (~ 180 mg/g cells/day) and constitutive (~ 30 mg/g cells/day) pathways into *Halomonas* generated production strains shown to be stable for up to 7 days.

**Conclusions:**

This study highlights new microbial pathways for the production of clean-burning bio-LPG fuels from amino acids. The use of stable *Halomonas* production strains could lead to gas production in the field under non-sterile conditions following process optimisation.

## Background

Global concerns about dwindling fossil fuel supplies have led to an urgent need to transition towards more sustainable bio-economy strategies [1]. This includes implementing economically viable routes to sustainable clean-burning biofuels derived from renewable waste biomass or industrial waste streams with minimal downstream processing costs. Microbial strategies for gaseous bio-alkane production (propane and butane) are promising because of the existence of well-established global markets (20 million tonnes propane per annum) and fuels distribution infrastructures [2]. The ‘drop-in’ nature of propane can boost the calorific value of existing biogas supplies, and propane has lower energy requirements for liquefaction and/or transportation [2]. In addition, the aerosol industry generates customer-specific gaseous hydrocarbon blends (propane, butane and isobutane) for use as propellants [3].

Engineered microbial routes to bio-propane and other gaseous hydrocarbons are now well established [4–9]. These pathways are dependent on variant forms of either aldehyde deformylating oxygenase (ADO) from *Prochlorococcus marinus* [4–6, 9, 10] or fatty acid photodecarboxylase (CvFAP) from *Chlorella variabilis* [4, 8, 11]. ADO variant A134F catalyses the NADPH-dependent decarbonylation of butyraldehyde to propane in the presence of its electron transfer partner ferredoxin [4, 6, 12]. CvFAP variants G462I and G462V were shown to catalyse the blue light-dependent decarboxylation of butyric and valeric acids to propane and butane, respectively [4]. In the latter case, microbial alkane production can be achieved in vivo by direct feeding of volatile fatty acid (VFA) precursor molecules. Alternative routes require the upregulation of butyraldehyde or butyric acid, utilising pathways derived from fatty acid biosynthesis [5], reverse β-oxidation [7], valine biosynthesis [9] or the clostridial butanol pathway [6].

**Fig. 1:**
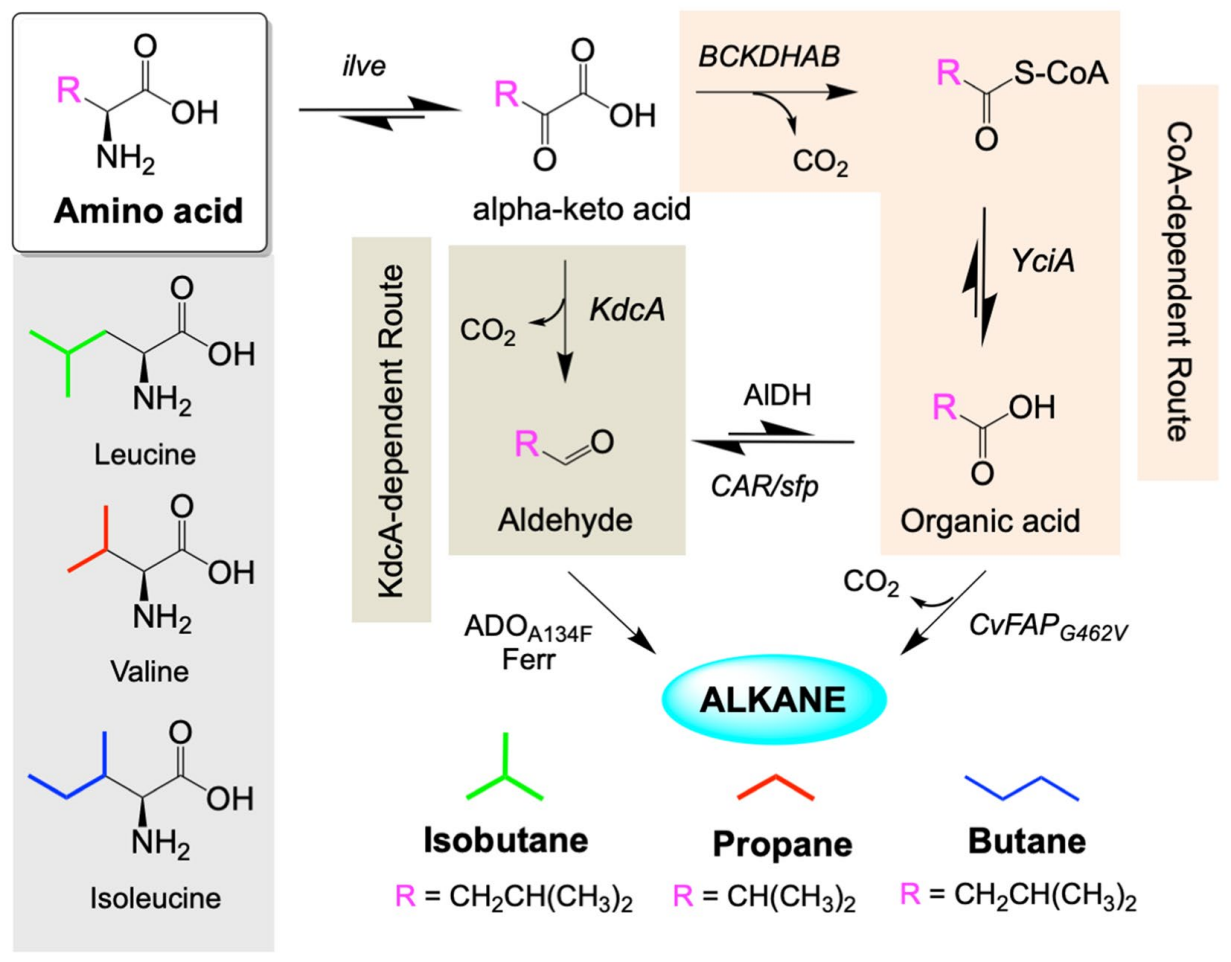
Overall scheme of the biocatalytic production of gaseous hydrocarbons propane, isobutane and butane from amino acids valine, leucine and isoleucine, respectively. Enzymes: ilvE = leucine 2-oxoglutarate transaminase from *E. coli*; BCKDHAB = human branched-chain α-keto acid dehydrogenase complex; YciA = acyl-CoA thioester hydrolase from *Haemophilus influenza*; CAR = carboxylic acid reductase from *Mycobacterium marinum*; sfp = maturation factor phosphopantetheinyl transferase from *Bacillus subtilis*; CvFAP = fatty acid photodecarboxylase from *Chlorella variabilis*; ADO = aldehyde deformylating oxygenase from *Prochlorococcus marinus*; Ferr = ferredoxin from *Synechocystis* sp. PCC6803 and KdcA = branched-chain keto acid decarboxylase from *Lactococcus lactis*. AlDH enzymes: αKGSDH = α-ketoglutaric semialdehyde dehydrogenase from *Burkholderia ambifaria*; PadA = phenylacetaldehyde dehydrogenase 17 from *E. coli*; or Hpad = 3-hydroxypropionaldehyde dehydrogenase puuC from *E. coli*

We recently explored the commercial potential of CvFAP-dependent bio-LPG production (bio-propane/butane blends) using recombinant *E. coli*, *Halomonas* and *Synechocystis* as microbial chassis [4]. Amino acids were explored as potential bio-alkane precursors, where valine (C3), isoleucine (*n*-C4) and leucine (*i*-C4) could be converted to propane, butane and isobutane, respectively (Fig. 1). In these pathways, amino acids undergo deamination by leucine 2-oxoglutarate transaminase (ilvE; [13]) to generate α-keto acids, followed by the action of branched-chain keto acid decarboxylase (KdcA; [14]) to generate the respective aldehyde. The action of the aldehyde dehydrogenase 3-hydroxypropionaldehyde dehydrogenase (Hpad; [15]) produced the equivalent carboxylic acid, which underwent decarboxylation to bio-alkane gas by CvFAP_G462I_ (Fig. 1). This KdcA-dependent route successfully produced bio-LPG blends in both *E. coli* and *Halomonas*, using proteinaceous waste as the source of amino acids [4].

In this study, we have explored the potential of additional CvFAP- and ADO-dependent routes to gaseous bio-alkanes from the amino acids valine, isoleucine and leucine. A second coenzyme A (CoA)-dependent [16] route was designed (Fig. 1; Additional file 1: Figs. S1, S2), and the original KdcA-dependent route was refactored to identify the optimal aldehyde dehydrogenase homologue and promoter system. Optimised pathways were incorporated into *E. coli* and the industrial chassis *Halomonas* either on plasmids or as stable chromosomally integrated strains. Fermentative production of gaseous bio-alkanes was achieved, demonstrating the potential of this approach for bio-LPG production. This study illustrates a potentially viable alternative to the VFA-fed CvFAP-dependent bio-alkane route for commercial exploitation, as food waste could act as both the bio-alkane precursor and carbon source, eliminating the need to supplement cultures with potentially cytotoxic VFAs.

## Results and discussion

### CoA-dependent pathway design from amino acids to bio-alkanes

A CoA-dependent (VFA-producing) pathway with CvFAP was designed, based on the ADO-dependent clostridial butanol pathway from butyryl-CoA to propane [6]. Similar to the KdcA pathway, an initial ilvE-dependent amino acid deamination generates the respective α-keto acids (Fig. 1). This is followed by CoA-dependent decarboxylation of the α-keto acid, catalysed by the human branched-chain α-keto acid dehydrogenase complex (BCKDHAB) [16]. CoA is subsequently eliminated by acyl-CoA thioester hydrolase (YciA; *Haemophilus influenza*) [6] to generate the respective carboxylic acid (Fig. 1). This is similar to the native leucine to isovaleric acid route described in *Propionibacterium freudenreichii* [17]. Finally, decarboxylative alkane production is catalysed by the G462V or G462I variant of CvFAP.

To generate the ADO-dependent pathway version, the carboxylic acid precursor is converted into the respective aldehyde by carboxylic acid reductase (CAR; *Mycobacterium marinum*) activated by the maturation factor phosphopantetheinyl transferase (sfp; *Bacillus subtilis*) [6]. The terminal step is the ADO- and ferredoxin-dependent decarbonylation of the aldehydes to bio-alkanes (Fig. 1). All of the enzymes in this pathway were shown to express in *E. coli* in an active form (Additional file 1: Figs. S3, S4; [4, 6]). An alternative CoA-dependent route from threonine to butane was also designed (Additional file 1: Figs. S2–S4), with the initial ilvE step substituted for threonine dehydratase (ilvA) from *E. coli* [18] and hydrocarbon chain extension performed by the *E. coli* leuABCD operon [19]. However initial constructs did not produce detectable propane due to the absence of activity of the LeuABCD-catalysed steps (results not shown). Therefore, the threonine-to-butane pathway was not investigated further in this study.

### CoA-dependent pathway bio-alkane gas production

**Fig. 2:**
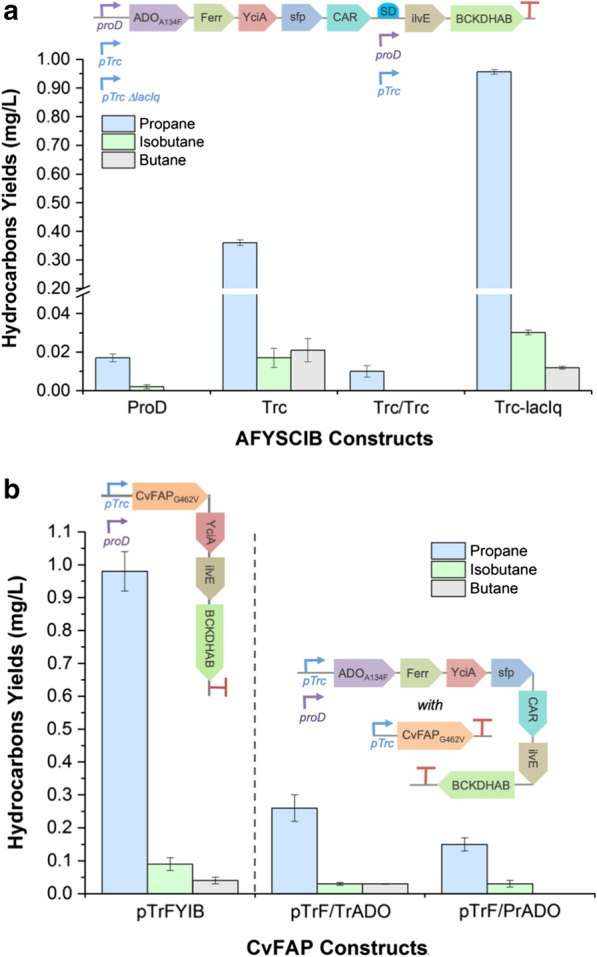
Hydrocarbon production of *E. coli* strain BL21(DE3)∆∆ containing engineered **a** ADO- and **b** CvFAP-dependant routes. Cultures (10 mL) were grown in LB medium containing 30 μg/mL kanamycin and 50 µg/mL chloramphenicol (pTrF/TrADO and pPrF/TrADO only) for 4–6 h at 37 °C and 180 rpm. Protein induction (100 μM IPTG) was performed for *trc*-containing constructs, and triplicate aliquots (1 mL) each of 3 biological replicate cultures were sealed into glass vials (3 mL) at 30 °C for 16–18 h at 200 rpm. For CvFAP-dependent constructs, the cultures were illuminated continuously with a blue LED (455 nm or 470 nm). Gaseous hydrocarbon levels were determined by manual headspace injection on an Agilent 490 Micro GC. Errors represent one standard deviation of the replicates (biological and/or technical triplicates). Constructs (inset): ProD = p*Pr*AFYSCIB; ProD/ProD = p*Pr*AFYSC*Pr*IB; Trc = p*Tr*AFYSCIB; Trc/Trc = p*Tr*AFYSC*Tr*IB and Trc-lacIq = p*Tr*Δ*L*AFYSCIB

To test the performance of in vivo bio-alkane production from amino acids, a series of five ADO-dependent DNA expression constructs were assembled in *E. coli* (AFYSCIB; ADO_A134F_-Ferr-YciA-sfp-CAR-ilvE-BCKDHAB). These constructs varied by the presence of either one or two isopropyl β-d-thiogalactopyranoside (IPTG)-inducible (*trc*) or constitutive promoters (*proD* or *trc*Δlac) [20] located upstream of the ilvE and/or ADO_A134F_ genes (Fig. 2a inset). The constitutive promoter *trc*Δlac was generated by removing the repressor protein gene (*lacI*) and the operator region from a standard *trc* promoter. Initial in vivo trials were performed with the single *proD*-controlled construct, expressed in multiple *E. coli* strains. Highest gaseous bio-alkane production was observed in the double deletion strain BL21(DE3)ΔΔ [4, 5], with propane making up to 79% of the total hydrocarbon gas in the culture headspace (0.11 ± 0.01 mg/L propane; Additional file 1: Fig. S5 and Table S1). This strain was designed previously to reduce the competing pathway from butyraldehyde to butanol by knocking out the *E. coli* aldehyde reductases Ahr and YqhD [5]. It has been shown to increase in vivo propane titres in ADO-dependent pathways [5, 6], so was selected as the *E. coli* chassis for the remainder of this work.

In vivo alkane production by five AFYSCIB constructs in *E. coli* showed the highest bio-alkane titres when only one promoter (*trc* or *proD*) was present upstream of ADO_A134F_ (Fig. 2a; Additional file 1: Table S2). The single *trc*–containing construct showed a 21-fold increase in propane titres over the equivalent *proD*-containing version. The best performing construct contained a single *trc*Δlac promoter (0.96 ± 0.01 mg propane/L culture), with yields similar to those obtained with the ADO_A134F_-dependent clostridial butanol pathway from glucose (3.4 mg/L [6]). Low levels of butane and/or isobutane were also detected with the single promoter constructs, with levels up to 10% of the total alkanes (up to 0.02 ± 0.01 mg butane/L culture; Fig. 2a). These data show that the CoA-dependent route can successfully generate low levels of multiple gaseous alkanes under standard *E. coli* cultivation conditions in the absence of additional amino acid supplementation. However, a prior study showed an ADO-dependent reverse-β-oxidation biosynthesis pathway from glycerol generated ca 22-fold higher butane titres (0.46 mg/L [7]).

Additional CoA-dependent pathways to bio-alkanes were designed to simplify the pathway and substitute the catalytically slow ADO enzyme [12] for the light-dependent CvFAP_G462V_ variant enzyme [4]. This latter enzyme has a reported reaction quantum yield of greater than 80% [8]. Pathway modification required the elimination of the two terminal steps catalysed by CAR/sfp and ADO_A134F_/Ferr, as CvFAP directly acts on the carboxylic acid precursor (Fig. 1). This shortened pathway may have the added advantage of a lower overall cellular burden in *E. coli* as fewer recombinant genes are expressed.

We generated two CvFAP-dependent constructs (F_G462V_YIB; CvFAP-YciA-ilvE-BCKDHAB; Fig. 2b inset), which differed by the type of promoter upstream of CvFAP_G462V_ (*trc* or *proD*). Gaseous alkane production was seen in *E. coli* only in the *trc*-containing construct in the presence of blue light, at titres similar to those seen with the best AFYSCIB pathway (0.98 ± 0.06 mg/L culture; Fig. 2b; Additional file 1: Table S2). Propane titres were at least tenfold higher than butane or isobutane, which differs from the CvFAP-dependent KdcA route [4] where the dominant gas produced was isobutane (4.8-fold higher than propane). This is likely due to substrate preferences of KdcA and/or differences in the amino acid concentrations present in the culture medium [21].

The requirement for blue light in CvFAP-dependent pathways necessitates adaptations to traditional in vivo bacterial cultivation strategies, as prolonged exposure to blue light (380–500 nm) is known to decrease the viability of *E. coli* [22] and cause the photoinactivation of CvFAP [4, 23]. Therefore, dark cycles (light absence) might be required periodically during fermentation to ensure the maintenance of sufficient cell density and the regeneration of biocatalysts. As ADO-dependent pathways do not require light, we tested *E. coli* strains expressing CvFAP_G462V_ with AFYSCIB pathways for alkane gas production (Fig. 2b inset). However, in each case bio-alkane gas production was lower than what was obtained with the individual pathways alone (0.15–0.26 mg propane/L culture; Fig. 2b; Additional file 1: Table S2). This could potentially be due to the additional cellular burden of extra gene overexpression and/or the requirement of two antibiotics to maintain both plasmids.

Overall, we have demonstrated gaseous alkane production in *E. coli* using the CoA-dependent routes, although the levels are at best ~ 6.5-fold lower than the KdcA-dependent route with CvFAP [4]. Therefore, we focused our studies on improving the KdcA-dependent pathway, which utilises CvFAP in place of ADO. This route shows more promise as a potential industrially useful route to gaseous bio-alkanes from amino acids.

### KdcA-dependent route refactoring

The existing KdcA-dependent pathway (Fig. 1) is a four-gene construct (IHKF_G462I_; ilvE-Hpad-KdcA-CvFAP [4]) controlled by two *trc* promoters upstream of ilvE and KdcA. We targeted the aldehyde dehydrogenase (AlDH) and terminal decarboxylation (CvFAP) steps in this pathway to see if further improvements in bio-alkane yields could be obtained. The existing AlDH is 3-hydroxypropionaldehyde dehydrogenase (Hpad) from *E. coli*, which have known activity towards isovaleraldehyde, butyraldehyde and valeraldehyde [15]. Two alternative aldehyde dehydrogenase homologues were selected, based on their known substrate specificities for short chain aldehydes. The first was α-ketoglutaric semialdehyde dehydrogenase (αKGSDH) from *Azospirillum brasilense* as it displays a known broad specificity [24]. The second homologue is phenylacetaldehyde dehydrogenase 17 (PadA) from *E. coli*, which is involved in phenylalanine degradation [25]. Each of these AlDH genes expressed in soluble active form in *E. coli* (Additional file 1: Figures S3, S4). In addition, both G462I and G462V variants of CvFAP were incorporated to see which supports the highest levels of bio-alkane gas production in vivo.

**Fig. 3:**
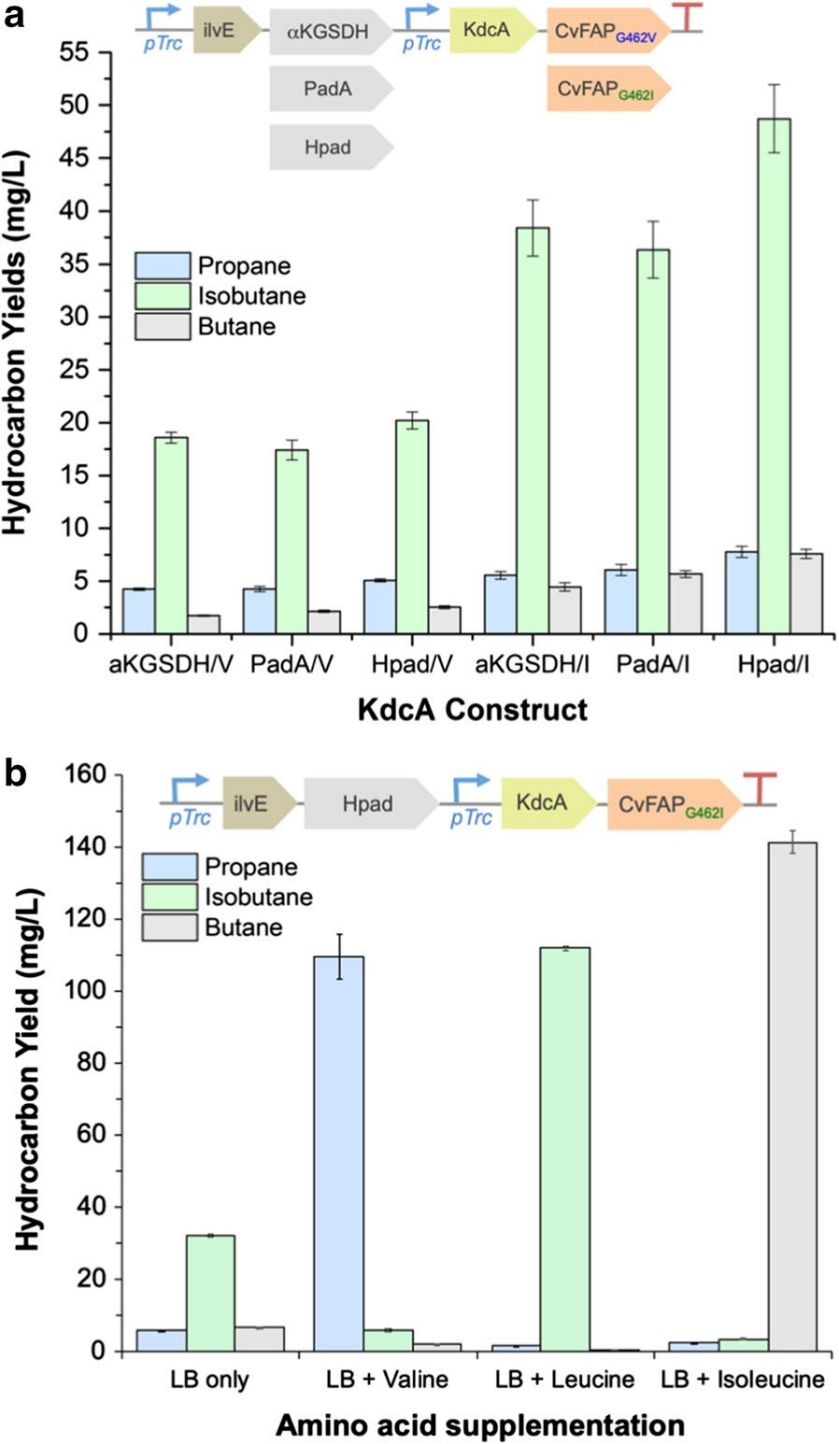
Hydrocarbons production of *E. coli* strain BL21(DE3)∆∆ containing engineered KdcA/CvFAP_G462V_-dependent routes. Gaseous alkane yields dependent on the **a** enzyme homologues and **b** amino acid supplementation for p*Tr*IH*Tr*KF_G462I_ construct. Cultures (10–20 mL) were grown in LB medium containing 30 μg/mL kanamycin for 4–6 h at 37 °C and 180 rpm. Luria broth contains ca 7, 8.8 and 5.4 mM valine, isoleucine and leucine, respectively [21]. Protein induction (100 μM IPTG) was performed, followed by culture supplementation with valine, leucine or isoleucine (30 mg/mL) in part B) after 1 h at 30 °C. Triplicate samples (1 mL) each of 3 biological replicate cultures were sealed into glass vials (4 mL) and incubated at 30 °C for 16–18 h at 200 rpm, illuminated continuously with a blue LED (455 nm or 470 nm). Gaseous hydrocarbon levels were determined by manual headspace injection on an Agilent 490 Micro GC. Errors represent one standard deviation of the replicates (biological and/or technical triplicates). Data for LB + valine was obtained from previous studies [4]. Expression constructs (inset): αKGSDH/V = *trc*-ilvE-αKGSDH-*trc*-kdcA-CvFAP_G462V_; Hpad/V = *trc*-ilvE-Hpad-*trc*-kdcA-CvFAP_G462V_; PadA/V = *trc*-PadA-Hpad-*trc*-kdcA-CvFAP_G462V_; αKGSDH/I = *trc*-ilvE-αKGSDH-*trc*-kdcA-CvFAP_G462I_; Hpad/I = *trc*-ilvE-Hpad-*trc*-kdcA-CvFAP_G462I_ and PadA/I = *trc*-PadA-Hpad-*trc*-kdcA-CvFAP_G462I_

Six KdcA-dependent pathways were constructed under *trc* control, differing by the AlDH homologue and the specific CvFAP variant (Fig. 3a inset). A shortened ADO-dependent version of this pathway (IKAF; ilvE-KdcA-ADO_A134F_-Ferr; Fig. 1) was not investigated in this study as prior studies suggested a CvFAP-dependent route is likely to be more productive [4]. For example, the CvFAP route is much more redox efficient as ADO needs a lot of cofactor, whereas CvFAP uses light for activation. In the CvFAP-dependent version the conversion of aldehyde back to acid by Hpad or homologues should also generate a reduced cofactor.

As expected, dramatically higher titres of gaseous bio-alkanes were detected in all six KdcA-dependent pathways compared to the CoA-dependent routes (Fig. 3a; Additional file 1: Table S2). In each case, the major gas produced was isobutane (73–79% total bio-alkane), with propane proportions decreased to only 12–18% of the total headspace alkane. In addition, there was a 2- to 2.4-fold increase in isobutane titres with CvFAP variant G462I over G462V [4]. There was a slight improvement in bio-alkane production with Hpad, consistent with its known specificity for isovaleraldehyde, butyraldehyde and valeraldehyde [15]. The highest bio-alkane-yielding construct generated isobutane titres of 48.7 ± 3.2 mg/L in standard *E. coli* growth medium. This is comparable to *E. coli* expressing CvFAP_G462I_ alone supplemented with isovaleric acid (87 mg/L culture; [4]). Production levels were also dependent on the plasmid backbone, as changing from a kanamycin-resistant to an ampicillin-resistant construct showed significant decreases in alkane titres (Additional file 1: Table S3).

The ratio of gases produced was similar in each KdcA-dependent pathway version, with isobutane:propane:butane ratios of ca 76:12:12 for the highest producing construct. This switch from predominantly propane to isobutane production suggests that KdcA may have a higher preference for α-ketoisocaproate over α-ketoisovalerate or α-keto-β-methylvalerate (Additional file 1: Figs. S1, S2). As each AlDH-containing construct generated similar ratios of each bio-alkane gas, the product profile might in part be influenced by the amino acid composition of the medium and their relative uptake rates into the cell.

### Tuneable bio-alkane blends

Ideally, a route to bio-LPG based on microbial fermentation would be tuneable, to allow precise control of the relative ratios of propane:butane (± isobutane) to suit customer requirements. This was demonstrated successfully in *E. coli* expressing only CvFAP variants, where propane-to-butane ratios were dependent on the relative proportions of butyric and valeric acids fed to the cultures, respectively [4]. A similar system could be applied using the KdcA-dependent pathway by supplementing the culture with specific amino acid blends. This approach could be a suitable alternative to VFA feeding as amino acid supplementation does not have the pH and cytotoxicity effects seen when adding millimolar concentrations of organic acids to cultures. In addition, a localised anaerobic digestion plant [4] would not be required to generate VFAs from waste biomaterial.

Leucine and isoleucine supplementation of *E. coli* cultures expressing IHKF_G462I_ showed proportional increases in isobutane and butane, respectively, without reaching saturation levels (Fig. 3b; Additional file 1: Fig. S6 and Table S4). These data are similar to previous studies with valine supplementation, where increasing levels of propane were generated [4]. With 30 mg/mL individual amino acid supplementation, similar yields of propane, isobutane and butane were detected (110 ± 6 [4], 112 ± 0.6 and 142 ± 3 mg/L, respectively). In each case, the predominant gas was greater than 95% of the total headspace alkane produced. Therefore, the desired country- or customer-specific requirements for propane:butane:isobutane ratios (bio-LPG or aerosol propellants [3]) during alkane gas production could be achieved by enriching the culture medium with specific amino acid blends. However, ideally the chosen microbial chassis should be modified to increase in vivo production of the required amino acid precursors, enabling the required bio-alkane blends to be generated from the locally derived waste feedstock.

Successful commercialisation of this approach would require the utilisation of cost-effective feedstocks and amino acid supply, such as proteinaceous waste (e.g. food waste). One study found the amino acid content of 39 samples of vegetal and dairy product food waste from EU industrial agro-food systems had a valine content between 6.4 and 29.4 mg/g waste [26]. Therefore, this waste could potentially be utilised as both a carbon and amino acid source.

### *Halomonas* as industrial chassis

Economically viable scaled bio-production strategies can be difficult to achieve due to high capital and operating expenditures of typical fermentation systems. This includes energy intensive running costs such as sterilisation, aseptic growth conditions, complex monitoring systems and downstream processing. Environmental concerns include the use of vast quantities of clean water and the route of waste disposal. To address these concerns, CvFAP-dependent bio-LPG production from VFAs was recently demonstrated in the halophilic production host *Halomonas* [4]. This microbial chassis grows at high salinity (up to 20% w/v NaCl) and pH values (up to 12), allowing for cultivation in seawater, waste-water or recycled water without the need for costly sterilisation and aseptic conditions [27]. This allows *Halomonas* bioreactors to be constructed using low cost materials, such as plastics, ceramics and cement, with overall costs savings of ca 65% compared to conventional scaled *E. coli* cultivation [28].

Given our recent success in transitioning CvFAP-dependent VFA to propane production from *E. coli* to *Halomonas* [4], we decided to take a similar approach with the KdcA-dependent amino acid pathway. Preliminary IPTG-inducible and constitutively expressed KdcA-dependent pathways were introduced into *Halomonas*, using both plasmid-borne and chromosomal integration strategies [4]. Bio-propane production was successfully demonstrated in small scale in vivo batch cultivation using valine-supplemented medium [4]. However, the scalability of the process was not tested, and construct optimisation had not been performed for expression in *Halomonas*. To address this, we utilised the highest performing *E. coli* construct (IHKF_G462I_) to generate six *Halomonas*-specific plasmids, which differed only by the promoter system. An efficient IPTG-inducible MmP1 T7-like promoter (*T7L*) was initially employed [4], as standard pET vector-based viral systems do not function in *Halomonas* [29]. Following our success in re-purposing *trc* into two constitutive promoters for *E. coli* (*TrΔL* and *ΔL*; Additional file 1: Table S5), we performed equivalent truncations in *T7L* to generate constitutive promoters *T7LΔL* and *HΔL*, respectively. We also tested three variant *Halomonas* promoters (*c102*, *c69* and *c59*), which are based on the major outer membrane protein porin constitutive expression system (Additional file 1: Fig. S11; [30, 31]). Preliminary studies with the constructs containing *T7L* and *c69* promoters in standard (non-supplemented) media were reported elsewhere [4], and form the basis of comparison with the remaining four constructs under constitutive control in this study.

**Fig. 4:**
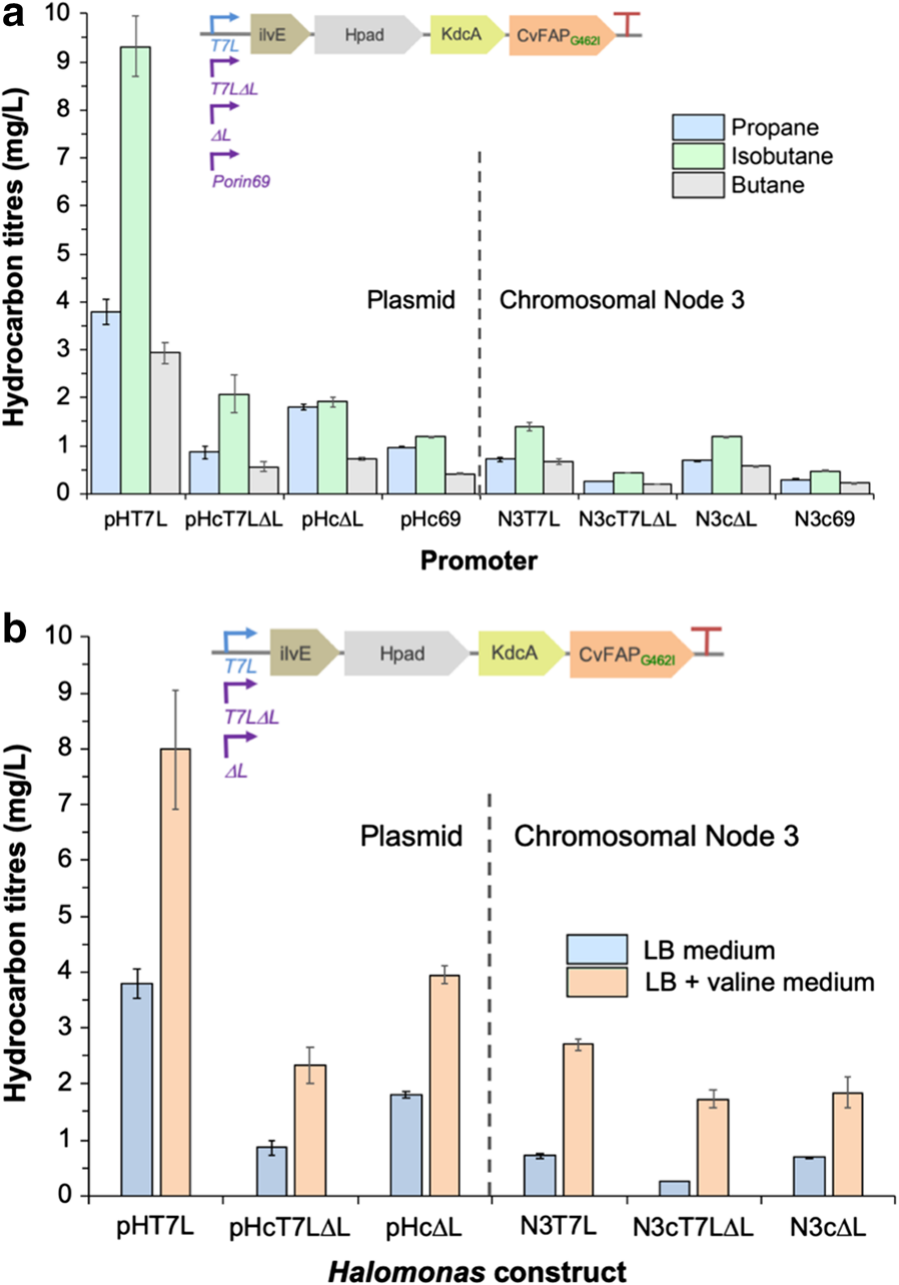
Effect of the KdcA-dependent pathway promoter on gaseous alkane production in *Halomonas* TQ10 in the **a** absence or **b** presence of valine culture supplementation. Pathway *promoter*-IH*Tr*KF_G462I_ was present as either a plasmid-borne or chromosomally integrated construct (single site). Cultures (20 mL) were grown in LB medium containing 30 µg/mL kanamycin for 4–6 h (OD_600_ ~ 0.6–0.8) at 37 °C and 180 rpm. Recombinant protein expression was induced with IPTG (0.1 mM) and culture supplementation with valine (30 mg/mL) after 1 h at 30 °C. Triplicate samples (1 mL) each of 3 biological replicate cultures were sealed into glass vials (4 mL) and incubated at 30 °C for 16–18 h at 200 rpm, illuminated continuously with a blue LED (455 nm or 470 nm). Gaseous hydrocarbon levels were determined by manual headspace injection using an Agilent 490 Micro GC, containing an Al_2_O_3_/KCl column. The errors represent one standard deviation of the data

The best performing construct in *Halomonas* contained the CvFAP_G462I_ variant under *T7L* control, generating primarily isobutane (9.32 ± 0.63 mg/L; Fig. 4a; [4]). This is around fourfold lower than the equivalent *E. coli* IHKF_G462I_ system under double *trc* control (38.6 ± 2.5 mg/L isobutane). Titres of propane were also decreased around twofold (3.78 ± 0.26 mg/L) in simple medium (no amino acid supplementation). For the constitutive expression systems, the best performers were controlled by *T7LΔL* and *HΔL* promoters (Fig. 4a; Additional file 1: Table S5). Surprisingly, titres of gaseous alkanes were lower using the *Halomonas*-specific *P*_*porin*_-like variant constitutive promoters (*c59*, *c69* and *c102*) compared to the truncated T7-like ones. In the majority of these constructs, the relative proportion of propane was increased relative to isobutane, to near equivalent levels.

The top three performers were further investigated by determining the increase in propane production in the presence of supplemental valine (30 mg/mL). Surprisingly, propane titres of the inducible construct only increased twofold (7.99 ± 1.07 mg/L; Fig. 4b; Additional file 1: Table S5), compared to the 17-fold increase with comparable constructs in *E. coli* (Fig. 3b; [4]). In each case, the relative concentrations of (iso)butane in valine-supplemented medium were dramatically decreased compared to standard media (Fig. 4b; Additional file 1: Table S5), likely due to competition for CvFAP active site. This suggests a limiting factor in this process may be the rate of amino acid uptake and/or endogenous amino acid metabolism by *Halomonas* compared to *E. coli*, and may also be contributing to the overall lower gaseous alkane titres in *Halomonas* compared to *E. coli*. Alternatively, one or more of the pathway enzymes may be unstable or poorly expressed in *Halomonas*. Notwithstanding, the prototype production pathway has been successfully demonstrated in *Halomonas*.

### Chromosomal integration into *Halomonas*

Chromosomal integration of pathways into an industrially robust microbial chassis is preferable to maintaining the genes for these pathways on a plasmid, as it eliminates the need for expensive and/or toxic selection agents [32], and prolongs the fermentation time to maximise product yields. To investigate this, we integrated four versions of the IHKF_G462I_ pathway into *Halomonas* strain TQ10 at two chromosomal loci (nodes 3 and 15; [33]), each differing only by the promoter type (*T7L, T7LΔL*, *HΔL* and *c69*). This was performed by utilising a novel suicide vector protocol (Additional file 1: Fig. S7), which was based on previously published methods [4, 34, 35]. Small-scale batch cultures were compared for gaseous alkane production in the presence and absence of supplemental valine (30 mg/mL).

As expected, the highest performing strain contained the inducible *T7L* promoter (1.39 ± 0.09 mg/L isobutane; [4]), however similar titres were obtained for the constitutive *HΔL* system (1.19 ± 0.01 mg/L isobutane; Fig. 4a; Additional file 1: Table S5). Overall, combined gas titres were significantly lower than for the equivalent plasmid systems, as in each case only a single copy was present in the genome. However, these differences were less marked in the presence of valine supplementation, with the *HΔL* construct showing only about twofold decrease in propane titres compared to the equivalent plasmid-borne system (1.84 ± 0.28 mg/L propane; Fig. 4b).

The chromosomal integration site had a significant impact on the overall gas titres, with loci 1 (node 3; [33]) showing ca a 2.5-fold increase over loci 2 (node 15; Additional file 1: Table S5). This suggests there is scope to significantly improve bio-gas titres by screening a variety of integration sites and/or multi-copy insertion of the construct into the *Halomonas* chromosome [33].

### Fermentative amino acid to bio-alkane production in *Halomonas*

**Fig. 5:**
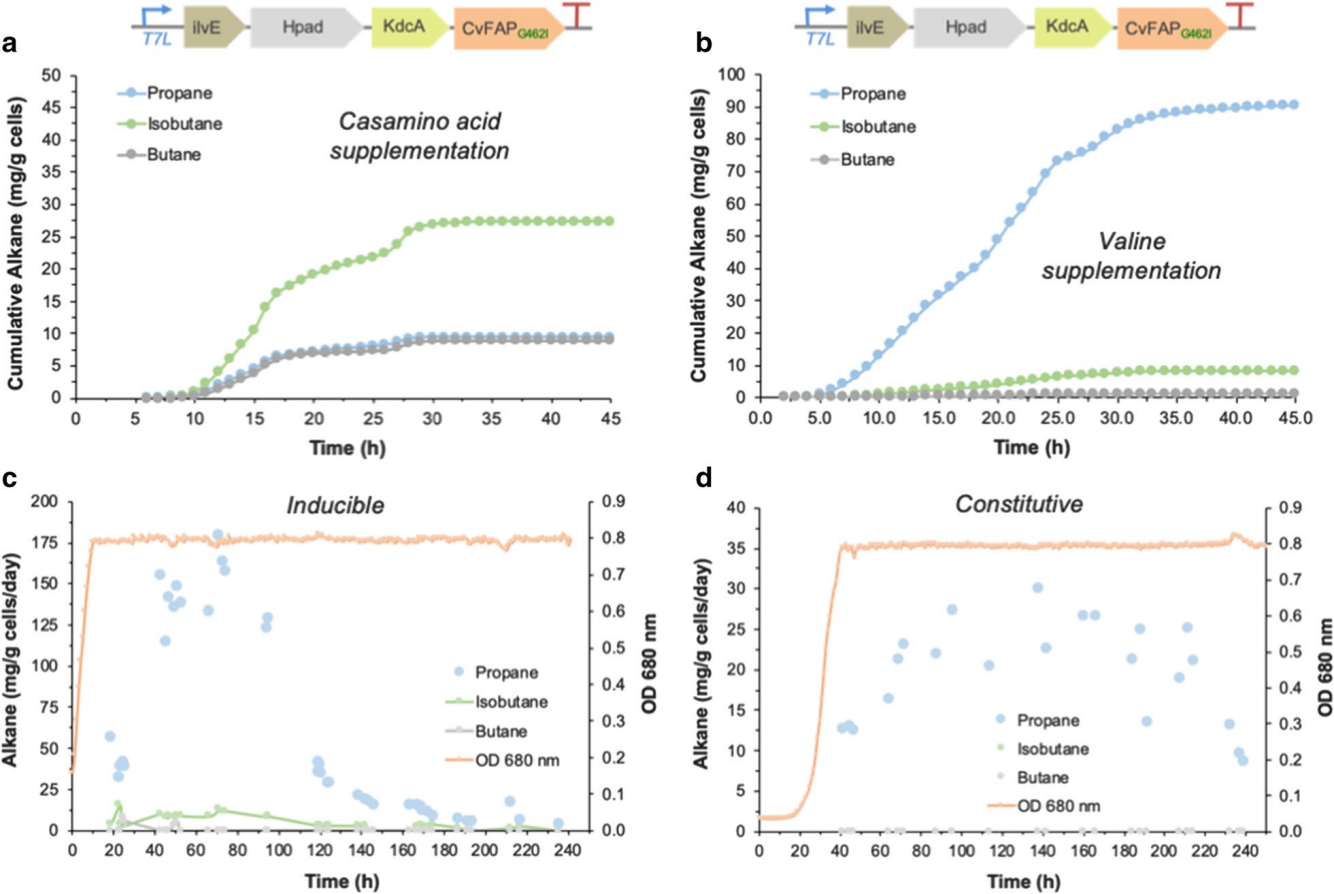
Bio-alkane production by *Halomonas* TQ10 expressing IHKF_G462I_ in plasmid or chromosomally integrated constructs. Cumulative alkane production by the plasmid-borne inducible construct pH*T7L*IHKF_G462I_ in LB60 medium supplemented with **a** casamino acids or **b** valine. Rate of alkane production of *Halomonas* TQ10 expressing chromosomally integrated **c** N3*T7L*IHKF_G462I_ (inducible) or **d** N3*cΔL*IHKF_G462I_ (constitutive). Photobioreactor cultivation (400 mL) was performed with medium pH 6.8 containing 0.5 mL/L antifoam and antibiotic (50 μg/mL spectinomycin or 34 μg/mL chloramphenicol for plasmid-borne and constitutive constructs, respectively). The culture was maintained at 30 °C with 60% stirring output with an airflow rate of 1.21 L/min, and ambient room lighting until mid-log phase (4–5 h). Protein expression was induced by 0.1 mM IPTG, where required, and the culture was maintained for 24–240 h with blue light exposure (1656). Culture medium feeding was employed to maintain an optical density of 0.8 and to replenish the carbon source for the chromosomal constructs. Alkane gas production was monitored by automated (**a**, **b**) or manual (**c**, **d**) headspace sampling using a Micro GC. The effect of amino acid supply on gaseous alkane titres was investigated by performing triplicate fermentations in three different culture media (Additional file 1: Figs. S8–10). Cumulative propane, isobutane and butane titres were calculated from the average production rates per hour in fermentations from freshly transformed *Halomonas* TQ10 in each media (**a**, **b** and Additional file 1: Fig. S11)

To investigate the scalability of this fermentative bio-alkane approach, we performed laboratory-scale cultivations of *Halomonas* expressing the plasmid-borne inducible IHKF_G462I_ construct (*T7L* promoter) up to 48 h in a flatbed photobioreactor (400 mL). Fermentations in standard *Halomonas* medium (~ 0.25% amino acid blend) containing glycerol [4] showed primarily propane production, with an estimated 17 mg propane/g cells over 48 h (Additional file 1: Fig. S11). The addition of casamino acids (~ 2% amino acid blend) led to predominantly isobutane production (max 27 mg propane/g cells), with propane and butane titres around threefold lower (about 9 mg propane/g cells; Fig. 5a). This switch in bio-alkane gas predominance is consistent with the individual amino acid concentrations in each medium (Additional file 1: Fig. S12). Valine-supplemented medium (1.8%) saw propane titres rise to around 90 mg propane/g cells in 46 h (Fig. 5b), equivalent to *Halomonas* TQ10 expressing CvFAP_G462V_ with supplemental butyric acid (89 mg propane/g cells; [4]). In most cases, the rate of gaseous alkane production peaked within the first 24 h, followed by a rapid decline in production (Additional file 1: Figs. S8–S10). This is attributed to plasmid instability and/or loss, or possibly CvFAP light inactivation [4, 23].

The longevity of the bioprocess was investigated by continuous flow fermentation of the genome integrated IHKF_G462I_*Halomonas* strains (*T7L* and *HΔL*; node 3) in valine-supplemented medium. Fermentation of the inducible (*T7L*) strain showed a gradual increase in propane production, peaking at around 70 h (~ 180 mg/g cells/day), followed by a slow decline to minimal titres by ~ 140 h (Fig. 5c). This suggests a gradual decrease in biocatalytic enzyme production, likely due to degradation/inactivation of IPTG and/or CvFAP photoinactivation [23] during the extended fermentation time [36]. Significant propane production in this strain was extended to nearly 5 days, however elimination of the need for IPTG could potentially extend propane production even further.

The constitutively expressed (*HΔL*) *Halomonas* strain showed a fairly consistent propane production rate from 60 to 200 h (up to 30 mg/g cells/day; Fig. 5d), followed by a rapid decline in bio-alkane production and cell viability. This loss of *Halomonas* viability is presumably due to the extended exposure to high-intensity blue light that is required for CvFAP activity. Cytotoxicity of blue light is a common feature of microorganisms in general [37]. Therefore, process optimisation to reduce the blue light intensity is required for efficient photocatalysis by CvFAP to extend propane production further. An alternative strategy could be employed where periodic ‘dark cycles’ are implemented, to allow cultures to regenerate to high cell density in the absence of light. For example, *Halomonas* TD01 (precursor of strain TQ10) is known to grow to high cell density (40 g/L cells dry weight) in 24 h with nutrient feeding [27]. This could be followed by feeding with amino acids and blue light exposure to switch on alkane gas production, until a decline in productivity is seen.

Overall, propane production in the constitutive strain decreased nearly eightfold from the equivalent IPTG-inducible strain. However, stable expression levels over an extended fermentation time could potentially generate higher levels of propane overall. Therefore, this fermentative approach from amino acids to gaseous alkanes could potentially be scaled to produce bio-LPG from proteinaceous feedstocks. Process and chassis engineering to increase bio-LPG titres could provide a viable alternative approach to the simpler route from VFAs.

## Conclusions

The fermentative KdcA-dependent pathway from amino acids to gaseous alkanes provides an alternative, yet complementary solution to VFA-dependent bio-LPG production. This wholly biological approach is in stark contrast to existing commercial ‘bio-LPG’ production strategies [38], which are essentially a chemically synthesised product from biologically sourced materials. Utilising *Halomonas* as the microbial chassis of choice, cultivated on renewable proteinaceous waste, could potentially reduce the process costs to enable successful commercialisation. Site-specific implementation of direct (VFA) or pathway driven (amino acid) strategies would be dependent on the availability and relative composition of local waste feedstocks, and the economics of waste pre-processing (hydrolysis vs anaerobic digestion). These renewable and sustainable approaches to clean-burning gaseous fuels could reduce the dependence on and environmental impact of fossil fuel combustion, and tap into the existing global market (US$19.5 billion in 2017) of amino acids.

## Materials, services and equipment

All chemicals, solvents and reagents were purchased from commercial suppliers, and were of analytical grade or better. The gas calibration standard was a custom blend of 1% each of propane, butane and isobutane in nitrogen (Thames Restek, Saunderton, UK). Media components were obtained from Formedium (Norfolk, UK). The *E. coli* strains used for propagating all plasmids were Stellar™ (Clontech) or NEB^®^5α (New England Biolabs). Expression studies were carried out using *E. coli* strains BL21(DE3) and NiCo21(DE3) (New England Biolab). *E. coli* strain BL21(DE3) was modified by chromosomal deletion of two aldehyde reductase genes *yqhD* and *yjgB* (BL21(DE3)∆yqhD/∆yjgB/Kan^R^) [5], and the kanamycin selection gene was removed (BL21(DE3)ΔΔ) as described previously [4]. *Halomonas* strain TQ10-MmP1 and modified pSEVA plasmids have been described previously [4].

Gene sequencing and oligonucleotide synthesis were performed by Eurofins MWG (Ebersberg, Germany). Details of all the plasmid and chromosomal constructs used in this study are found in Additional file 1: Tables S5–S7. pSBR1Ks-i-SceI is a hybrid of pSEVA and Biobrick (pBb-type) plasmids, containing oriT, I-SceI meganuclease gene under *trc* control, pRO1600-ColE1 double origin and both kanamycin and spectinomycin resistance markers. Plasmids pSH-N3 and pSH-N15 are based on the CRISPR/Cas9 editing *Halomonas* genome donor DNA pSEVA241 plasmid of Quin et al. [35], except the gRNA and antibiotic resistances were removed, and the insert contained the target DNA with a pKIKO-derived chloramphenicol resistance gene flanked by FRT sequences [39]. Node 3 and node 15 refer to *Halomonas* chromosomal loci for insertion of target DNA [33]. The modified pPorin-like constitutive promoter plasmids pH*c102*-RFP, pH*c69*-RFP and pH*c59*-RFP were kindly supplied by Duangthip Trisrivirat (Vidyasirimedhi Institute of Science and Technology; VISTEC). These plasmids are a modified pHal2-based plasmid [4] containing RFP with the T7-like promoter swapped for a modified pPorin-like constitutive promoter. The BglBrick series of vectors were obtained from Addgene (https://www.addgene.org) [40]. Gene synthesis was performed by GeneArt (ThermoFisher, Germany) or GenScript (USA). The sequences of all the oligonucleotides used in cloning and mutagenesis can be found in Additional file 1: Tables S8–S12.

The mounted high-power blue LEDs and LED drivers were from Thorlabs (Ely, U.K.), with spectra centred at 470 nm (FWHM 25 nm, 710 mW typical output). The custom-built LED blue light array had area of 396 cm^2^ of relatively consistent light intensity and a fixed average culture-to-LED distance of 8 cm. The photobioreactor was a thermostatic flat panel FMT 150 (500 mL; Photon Systems Instruments, Czech Republic) with integral culture monitoring (OD 680/720 nm), pH and feeding control and an LED blue light panel (465 nm; maximum PPFD = 1648 μE photons).

### Single and multi-gene construct synthesis

The propane synthesis plasmids pTPC7 (YciA-sfp-CAR; [6]), ADO_A134F_, petF (Ferr) and pCvFAP_G462V_ (fatty acid photodecarboxylase from *Chlorella variabilis*) were assembled as described previously [4, 6]. A second IPTG-inducible CvFAP_G462V_ construct was generated by sub cloning the variant gene into pBbA1c-RFP (p*Tr*CvFAP_G462V_), eliminating the RFP gene. The following genes were designed, synthesised and sub-cloned into pET21b, incorporating a C-terminal His_6_-tag: leucine 2-oxoglutarate transaminase from *E. coli* (ilvE; UniProt: P0AB80); human branched-chain α-keto acid dehydrogenase (BCKDHAB; P12694 and P21953; His_6_-tag on subunit B only), phenylacetaldehyde dehydrogenase 17 from *E. coli* (PadA; P80668), threonine dehydratase from *E. coli* (ilvA; P04968) and the *E. coli* leuABCD complex composed of 2-isopropylmalate synthase (LeuA; P09151), 2-isopropylmalate dehydrogenase (LeuB; P30125) and isopropyl malate isomerase complex (LeuC/LeuD; P0A6A6/P30126). Additional synthesised genes sub-cloned into pETM11 were α-ketoglutaric semialdehyde dehydrogenase from *Azospirillum brasilense* (αKGSDH; Q1JUP4) and 3-hydroxypropionaldehyde dehydrogenase from *E. coli* (Hpad; P23883), while branched-chain keto acid decarboxylase from *Lactococcus lactis* (KdcA; Q6QBS4) was sub cloned into pET28b. These latter genes contained a vector-derived N-terminal His_6_-tag. Genes were codon optimised to remove rare codons for optimal expression in *E. coli*. For LeuABCD, the native *E. coli* operon sequence was synthesised (no His_6_-tags), with gene expression controlled by a single T7 promoter (Additional file 1: Table S5).

The multi-gene construct pYSCAP (YciA-sfp-CAR-ADO_A134F_-Ferr) contained the genes encoding non-His_6_-tagged versions of acyl-CoA thioester hydrolase from *Haemophilus influenza* (YciA; P44886); maturation factor phosphopantetheinyl transferase from *Bacillus subtilis* (sfp; P39135); carboxylic acid reductase from *Mycobacterium marinum* (CAR; B2HN69); aldehyde deformylating oxygenase variant A134F from *Prochlorococcus marinus* (ADO; Q7V6D4) and ferredoxin from *Synechocystis* sp PCC6803 (Ferr; P27320) [5, 6]. This construct was synthesised as a complete operon with codon-optimised genes, synthetic Shine–Dalgarno (SD) sequences and the constitutive promoters R0011 (http://2015.igem.org/) and proD upstream of YciA and ADO_A134F_, respectively [20].

### Multi-gene constructs assembly in *E. coli*

In most cases, the assembly of multi-gene constructs was performed by In-Fusion cloning, according to the manufacturer’s protocols [41]. Vector linearisation and insert(s) amplification were performed by  polymerase chain reaction (PCR), using the CloneAmp™ HiFi PCR Premix kit (Clontech), incorporating 15–25 bp overhangs necessary for subsequent ligations. In some cases, overlap extension PCR (OEP) was performed to ligate two or more DNA fragments generated by PCR to simplify subsequent construct assembly. In this method an initial 5 PCR cycles were performed with the template DNA fragments only, followed by the addition of the forward primer of the first DNA fragment and the reverse primer of the last insert. Following In-Fusion cloning, each construct was transformed into the *E. coli* strain Stellar or NEB5α for plasmid recovery, and the correct assembly was confirmed by DNA sequencing. The oligonucleotide sequences and template DNA used in each PCR reaction is shown in Additional file 1: Tables S8–S12. The following sections will detail the general approaches taken for the assembly of each plasmid.

#### ADO-containing CoA-dependent multi-gene *E. coli* constructs assembly

A dual construct was assembled in pBbE2k [40] containing ADO_A134F_ and its electron transfer partner Ferr [6], under the control of a tetracycline-inducible promoter. Both ADO_A134F_ and Ferr genes were PCR amplified from their respective constructs in pCDFDuet-1 and pRSF-Duet1 [6]. The PCR products included the existing vector-derived 5′-SD sequence for ADO_A134F_ and a non-native SD sequence (GGAGGACAGCTAA) for Ferr. In-Fusion cloning was performed with the linearised destination vector pBbE2k-RFP, minus the RFP gene and its SD sequence, to generate p*Tet*ADO_A134F_Ferr (Additional file 1: Table S8).

The assembly of a butyryl-CoA to propane pathway construct pAFYSC (*T7*-ADO_A134F_-Ferr-YciA-sfp-CAR) was performed using the vector pETDuetT-1. PCR linearisation of construct TPC7 (*T7*-YciA-sfp-CAR) occurred between the T7 promoter and the initial SD sequence, while the two genes from pADO_A134F_Ferr were amplified with both SD sequences. In-Fusion cloning between the two PCR products generated the IPTG-inducible pAFYSC pathway (Additional file 1: Table S8).

To eliminate the need for IPTG induction, a constitutive expression system was constructed using the BglBrick plasmid pBbE7k-RFP as the backbone [40]. The plasmid was linearised by reverse PCR, eliminating the T7 promoter-RFP cassette (*lac*I^q^ retained), and the *proD*-ADO_A134F_ insert was amplified from the multi-gene construct pYSCAP. Following In-Fusion cloning, the new ADO-containing constitutive expression vector (p*Pr*ADO_A134F_) was used as the backbone for the construction of a series of ADO-dependent pathways from butyryl-CoA to propane. The first constitutive pathway assembled was pPr*AFYSC (proD*-ADO_A134F_-Ferr-YciA-sfp-CAR), via the ligation of the pAFYSC pathway genes (SD-ADO_A134F_-Ferr-YciA-sfp-CAR) into the linearised proD-containing empty plasmid (p*Pr*ADO_A134F_ minus SD-ADO_A134F_; Additional file 1: Table S8). This was followed by linearisation of pPr*AFYSC by reverse PCR to eliminate the now redundant lacI^q^ repressor. The new construct (p*Pr*AFYSC) was re-circularised by In-Fusion cloning in the absence of any insert.

The generation of ADO-dependent pathways to propane, butane and isobutane from the amino acids valine, isoleucine and leucine, respectively, requires the addition of genes ilvE and BCKDHAB to the existing AFYSC constructs (Fig. 1). Initially a constitutively controlled dual enzyme construct (pPr*IB; proD*-ilvE-BCKDHAB) was assembled in the same modified BioBrick plasmid as used for pPr*AFYSC construction. The individual genes (ilvE and BCKDHAB) were amplified by PCR from their respective synthesised constructs. This was followed by OEP to generate a dual enzyme insert, followed by ligation to the linearised empty vector (pPr*AFYSC minus AFYSC). A complete pathway from amino acid to gaseous hydrocarbon was generated by the inclusion of ilvE-BCKDHAB with AFYSC to form pPr*AFYSCIB (proD*-ADO_A134F_-Ferr-YciA-sfp-CAR-ilvE-BCKDHAB). This was performed by linearising p*Pr*AFYSC after CAR, and ligating it to the ilvE-BCKDHAB insert amplified from pPr*IB. To increase the expression of ilvE and BCKDHAB, a second *proD* promoter was inserted downstream of CAR in p*Pr*AFYSC by In-Fusion cloning, to generate p*Pr*AFYSC*Pr* (Additional file 1: Table S8). Insertion of the ilvE-BCKDHAB fragment of pPr*IB after the second *proD* generated pPr*AFYSC*Pr*IB (proD*-ADO_A134F_-Ferr-YciA-sfp-CAR-*proD*-ilvE-BCKDHAB.

IPTG-inducible constructs catalysing gaseous hydrocarbon production from amino acids were generated by amplifying the seven pathway genes from pPr*AFYSCIB and ligating them into pBbE1k, linearised downstream of the *trc* promoter (p*Tr*AFYSCIB; *trc*-ADO_A134F_-Ferr-YciA-sfp-CAR-ilvE-BCKDHAB). Similarly, the addition of a second *trc* promoter upstream of ilvE was performed by PCR coupled to In-Fusion cloning to generate p*Tr*AFYSC*Tr*IB (*trc*-ADO_A134F_-Ferr-YciA-sfp-CAR-*trc*-ilvE-BCKDHAB; Additional file 1: Table S8).

#### FAP-containing CoA-dependent multi-gene *E. coli* constructs assembly

Simplified gaseous hydrocarbon producing constructs from amino acids were generated by substituting the four genes CAR, sfp, ADO_A134F_ and Ferr for a single gene CvFAP variant G462V or G462I. An initial construct p*Tr*F_G462V_YIB was generated (*trc*-CvFAP_G462V_-YciA-ilvE-BCKDHAB) using p*Tr*AFYSCIB as the backbone. This latter plasmid was linearised, eliminating the genes encoding AFYSC (Additional file 1: Table S9). A dual gene insert was constructed (CvFAP_G462V_-YciA) by PCR amplification of each individual gene, followed by OEP. This was ligated to the backbone plasmid upstream of ilvE, generating an IPTG-inducible construct. A similar constitutive construct p*Pr*F_G462V_YIB (*proD*-CvFAP_G462V_-YciA-ilvE-BCKDHAB) was generated as above, except the backbone template was the *proD*-containing plasmid p*Pr*AFYSCIB.

#### KdcA-dependent multi-gene *E. coli* constructs assembly

Alternative CvFAP_G462V_-dependent pathways were constructed by the substitution of six genes (BCKDHAB, YciA, CAR, sfp, ADO_A134F_ and Ferr) for KdcA, an alcohol dehydrogenase and CvFAP_G462V_. These pathways were constructed in pBbE1k, including the insertion of a second *trc* promoter upstream of the latter two genes (*trc*-KdcA-CvFAP_G462V_). Each individual gene and second *trc* promoter were amplified by PCR, and OEP was performed between *trc* and KdcA DNA fragments (Additional file 1: Table S10). In-Fusion cloning was performed generating three KdcA-dependent and IPTG-inducible constructs (p*Tr*IA*TrKF_G462V_, where A* = αKGSDH, PadA or Hpad; Additional file 1: Table S5). Each of these three constructs underwent site-directed mutagenesis of the CvFAP gene to produce the equivalent pathway with the variant G462I (p*Tr*IαK*Tr*KF_G462I_, p*Tr*IΠ*Tr*KF_G462I_ and p*Tr*IΗ*Tr*KF_G462I_, respectively) as described previously [4].

### *Halomonas* KdcA-dependent construct assembly

Six KdcA-dependent pathways were constructed in the *Halomonas*-compatible plasmid pHal2 [4], which varied by the type of promoter used. This was performed by multi-step In-Fusion cloning, where PCR was used to amplify the inserts, eliminating the His_6_-tags, and/or linearise the vectors (Additional file 1: Table S11). Each construct was propagated in the *E. coli* conjugative donor strain S17-1 [42]. Plasmid transformation into *Halomonas* was performed by conjugation according to the method described previously [4]. Plasmid content of each trans-conjugant was confirmed by DNA isolation, restriction mapping and sequencing.

The initial construct was generated under control of the IPTG-inducible MmP1 T7-like promoter (pH*T7L*IHKF_G462I_; [4]), which later underwent LacI^q^ elimination (*T7LΔL*) to generate the respective constitutive construct (pH*T7LΔL*IHKF_G462I_). A second constitutively expressed construct was generated by substituting the T7-like promoter for a truncated *trc* promoter, which was deficient in both *trc* and *lacI* (pH*ΔL*IHKF_G462I_). Three constitutive promoters were generated (*c102*, *c69* and *c59*) based on the major outer membrane protein porin constitutive expression system in *Halomonas* (Additional file 1: Fig. S11; [30, 31]). The IHKF_G462I_ operon was inserted downstream of each promoter, generating a further three *Halomonas* constructs (pH*c102*IHKF_G462I_, pH*c69*IHKF_G462I_ and pH*c59*IHKF_G462I_).

### Chromosomal integration of pathway genes into *Halomonas*

Chromosomal insertion of the KdcA-dependent pathways into *Halomonas* TQ10 was performed using a novel suicide vector (pSH) protocol (Additional file 1: Fig. S7) based on previously published methods [4, 34, 35]. The pSH insertion plasmids (pSH-N3 and pSH-N15) contained the biocatalytic and pKIKO-derived FRT flanked [39] chloramphenicol resistance genes surrounded by homology arms (node 3 or 15; [33]), an I-SceI restriction site and a *colE1* ori (incompatible) with replication in *Halomonas* (Additional file 1: Fig. S10 inset). This plasmid was co-conjugated into *Halomonas* TQ10-MmP1 with a second spectinomycin-resistant plasmid (pSBR1Ks-i-SceI), the latter expressing the restriction enzyme I-SceI. In vivo expression of I-SceI linearised pSH plasmids facilitates chromosomal integration [34, 35]. Successful integration was seen as growth of *Halomonas* on chloramphenicol-selective medium, as the pSH plasmid is not replicated in *Halomonas*. Integration was confirmed by colony PCR, genomic sequencing and in vivo propane production after pSceI plasmid curing [34, 35].

Eight KdcA-dependent constructs (ilvE-Hpad-kdcA-CvFAP_G462I_) were integrated into *Halomonas* strain TQ10 (Additional file 1: Table S12). The constructs varied by the chromosomal loci (node 3 or 15) and the promoter type (inducible vs constitutive). The inducible system was the MmP1 T7-like promoter (*T7L*), while the constitutively expressed constructs were controlled by pPorin-like 69 (*c69*), lacI^q^-deficient MmP1 T7-like promoter (*T7LΔL*) or the truncated pTrc promoter minus *trc* and *lacI* (*ΔL*; Additional file 1: Table S12). The insertion of inducible *T7L*IHKF_G462I_ or constitutive *c69*IHKF_G462I_ constructs into pSH-N3 or pSH-N15 was performed via In-Fusion cloning using PCR linearised destination vectors (between one homology arm and upstream of the chloramphenicol gene; Additional file 1: Fig. S5) and amplified multi-gene constructs with their own promoters (N3- or N15*T7L*IHKF_G462I_; N3- or N15*c69*IHKF_G462I_). For the *ΔL*-containing plasmids, a similar protocol was performed as above, except the *T7L*IHKF_G462I_ construct for each node was used as the template, and the only DNA eliminated/inserted was the promoter (N3- or N15*cΔL*IHKF_G462I_). To generate the constructs with the *T7LΔL* promoter, PCR elimination of the *laci*^*q*^ gene was performed on the equivalent *T7L*-containing constructs followed by self In-Fusion cloning to re-circularise the plasmid (N3- or N15*cT7LΔL*IHKF_G462I_). Successful integration of the constructs at the correct loci was confirmed by colony PCR and genome sequencing.

### Protein expression and lysate production

IPTG-dependent expression of proteins YciA, CAR, sfp, CvFAP_G462V_, ADO_A134F_ and Ferr in *E. coli* has been demonstrated previously [4, 6]. The remaining proteins ilvE, BCKDHAB, KdcA, αKGSDH, PadA, Hpad ilvA and LeuABCD were transformed into *E. coli* strain BL21(DE3) for protein overexpression studies. Cultures (1 L) were grown in LB Broth Miller (Formedium) containing the required antibiotic (50 μg/mL ampicillin or 30 μg/mL kanamycin) at 37 °C with 180 rpm shaking until OD_600nm_ = 0.6. Recombinant protein induction was performed with IPTG (0.1 mM), followed by a further 12–16 h incubation at 25 °C. Cells were harvested by centrifugation at 3320×*g* for 30 min at 4 °C.

Cells were resuspended in lysis buffer (5 mL/g pellet; 50 mM Tris pH 7.0 containing 1 mM MgCl_2_, 1 mM β-mercaptoethanol, 10% glycerol, 2X protease inhibitors, 50 μg/mL DNAse and 50 μg/mL lysozyme) and freeze-thawed in liquid nitrogen. Cells were lysed by sonication, and clarified using centrifugation (48,000×*g*). Protein content was determined using 12% SDS-PAGE gels (Mini-Protean TGX Stain-Free Precast Gels, Bio-Rad). Protein gels were imaged using a BioRad Gel Doc EZ Imager and relative protein band intensity was determined using the BioRad ImageLab software. Identification of His_6_-tagged proteins was performed by Western blots using the Trans-Blot^®^ Turbo™ Transfer system (PVDF membranes; BioRad) and the Western Breeze Chemiluminescent Immunodetection kit (alkaline phosphatase; Life Technologies) with mouse (His tag monoclonal antibody) and alkaline phosphatase-containing (Anti-C-My) primary and secondary antibodies, respectively.

### Gaseous hydrocarbon production

In vivo gaseous hydrocarbon production by recombinant *E. coli* was performed using the following general protocol: cultures (10 mL) were incubated for 4–6 h (OD_600_ ~ 1.6–2) at 37 °C and 180 rpm in LB or TB medium containing 50 µg/mL ampicillin, 30 µg/mL kanamycin or 50 µg/mL chloramphenicol, dependent on the antibiotic resistance (Additional file 1: Table S5). Supplemental valine, leucine or isoleucine (up to 30 mg/L) were included in the medium where required. Protein induction (0.1 mM IPTG) was performed for *trc* or T7-containing constructs, and triplicate samples (1 mL) each of 3 biological replicate cultures were sealed into glass vials (3 mL) and incubated at 30 °C for 16–18 h at 200 rpm, illuminated continuously with a blue LED (455 nm or 470 nm).

For propane production in *Halomonas*, LB60 medium (1% tryptone, 0.5% yeast extract, 6% NaCl) pH 9.0 was used containing spectinomycin (50 μg/mL). Cultures were agitated (180 rpm) at 37 °C for 5 h incubation (OD ~ 1.6–2) prior to induction. IPTG (0.1 mM) was added (where necessary), and the remaining methodology was performed as described above for *E. coli* cultures.

### Photobioreactor cultivation

General photobioreactor cultivation (400 mL) was performed with high salt glycerol medium (30–32% seawater or Instant Ocean, NaCl to 6%, 0.1% glycerol and 0.5% yeast extract) pH 6.8 containing 0.5 mL/L antifoam and antibiotic (50 μg/mL spectinomycin or 34 μg/mL chloramphenicol for plasmid-borne and constitutive constructs, respectively). Alternative growth media were based on LB60 pH 6.8, which were supplemented with 1.5% valine (LB60Val) or casamino acids (LB60Cas). Cultivation was performed in batch mode, pre-equilibrated at 30 °C with 60% stirring output. An overnight starter culture (10–15 mL) of *Halomonas* TQ10 expressing pH*T7L*IHKF_G462I_ was added, to achieve a starting OD680 nm of ~ 0.2, and the culture was maintained at 30 °C with an airflow rate of 1.21 L/min, automated pH maintenance, culture optical density monitoring and ambient room lighting until mid-log phase (4–5 h). Protein induction by IPTG (0.1 mM) was performed for *T7L*-promoter systems with continual monitoring for 2–10 days with blue light exposure (1656 or 600 μE for plasmid-borne and chromosomal systems, respectively). Alkane gas production was monitored at 20-min intervals by automated headspace sampling using a Micro GC, while aqueous amino acid and glycerol depletion were quantified by HPLC.

Fermentations of *Halomonas* TQ10 containing chromosomally integrated N3*T7L*IHKF_G462I_ or N3*cΔL*IHKF_G462I_ was performed as above with LB60Val pH 6.8, except culture medium feeding was employed to maintain an optical density of 0.8 and to replenish the carbon source. Cultures were maintained for about 240 h, with alkane gas production monitored at 2–3 times daily by manual sampling using a Micro GC.

### Analytical techniques

Propane levels were determined by manual headspace injection or automated (fermentation off gas monitoring) using an Agilent 490 Micro GC, containing an Al_2_O_3_/KCl column and a thermal conductivity detector (TCD). Aqueous culture metabolites (VFAs and glycerol) were analysed by HPLC using an Agilent 1260 Infinity HPLC with a 1260 ALS autosampler, TCC SL column heater, a 1260 refractive index detector (RID) with an Agilent Hi-Plex H column (300 × 7.7 mm; 5 mM H_2_SO_4_). The running conditions for both the Micro GC and HPLC were the same as described previously [4]. For amino acid quantitation, analysis was performed according to the method of Bartolomeo and Maisano [43]. Each analyte concentration was calculated by comparing the peak areas to a standard curve generated under the same running conditions. Error bars indicate one standard deviation of the data obtained for the replicates (biological and/or technical triplicates).

## Supplementary information

**Additional file 1.** Additional Figures and Tables with additional data and methods.

## Data Availability

The datasets used and/or analysed during the current study are available from the corresponding author on reasonable request.

## Abbreviations

ADO: Aldehyde-deformylating oxygenase
BCKDHAB: Human 2-oxoisovalerate dehydrogenase α-and β-subunits
CAR: Carboxylic acid reductase
CvFAP: *Chlorella variabilis* fatty acid photodecarboxylase
Ferr: Ferredoxin
Hpad: 3-Hydroxypropionaldehyde dehydrogenase
ilvA: l-Threonine dehydratase biosynthetic
ilvE: Branched-chain amino acid aminotransferase
IPTG: Isopropyl β-d-1-thiogalactopyranoside
KdcA: Branched-chain keto acid decarboxylase
LB: Luria broth
LeuABCD: Isopropyl malate synthase, dehydrogenase and isomerases complex
LPG: Liquefied petroleum gas
PadA: Phenylacetaldehyde dehydrogenase
sfp: Maturation factor phosphopantetheinyl transferase
YciA: Acyl-CoA thioester hydrolase
αKGSDH: α-Ketoglutaric semialdehyde dehydrogenase
NADPH: Reduced nicotinamide dinucleotide phosphate
bio-LPG: Biologically sourced gaseous alkane gases
IPTG: Isopropyl β-d-thiogalactopyranoside
PCR: Polymerase chain reaction
DNA constructs: AFYSCIB: ADO_A134F_-Ferr-YciA-sfp-CAR-ilvE-BCKDHAB
F_G462V_YIB: CvFAP_G462V_-YciA-ilvE-BCKDHAB
IHKF_G462I_: ilvE-Hpad-KdcA-CvFAP_G462I_
IPTG: Isopropyl β-d-thiogalactopyranoside
