# Supplementary material for: Renewable and tuneable bio-LPG blends derived from amino acids
Source: Biotechnol Biofuels. 2020 Jul 14;13:125. doi: 10.1186/s13068-020-01766-0 (PMC7362463; doi:10.1186/s13068-020-01766-0)
Supplement: Supplementary file 1 — Additional file 1. Additional Figures and Tables with additional data and methods. [file 13068_2020_1766_MOESM1_ESM.docx]

# Additional Information

**Renewable and Tuneable Bio-LPG Blends Derived from Amino Acids**

**Mohamed Amer,^1^ Robin Hoeven,^1^ Paul Kelly,^1^ Matthew Faulkner,^1^ Michael H. Smith,^2^ Helen S. Toogood,^1^ and Nigel S Scrutton^1,2^***

^1^EPSRC/BBSRC Future Biomanufacturing Research Hub, BBSRC/EPSRC Synthetic Biology Research Centre SYNBIOCHEM Manchester Institute of Biotechnology and School of Chemistry, The University of Manchester, Manchester, M1 7DN, UK.

^2^C3 Biotechnologies Ltd, The Railway Goods Yard, Middleton-in-Lonsdale, Lancashire, LA6 2NF, UK.

**Supporting Figures**

**Figure S1**. Potential amino acid derived biological routes for the production of a) propane from valine and b) isobutane from leucine. Enzymes: ilvE = leucine 2-oxoglutarate transaminase from *E. coli*; BCKDHAB = human branched-chain α-keto acid dehydrogenase complex; LeuA = 2-isopropylmalate synthase from *E. coli*; LeuB = 2-isopropylmalate dehydrogenase from *E. coli*; LeuC/LeuD = isopropyl malate isomerase complex from *E. coli*; YciA = acyl-CoA thioester hydrolase from *Haemophilus influenza*; CAR = carboxylic acid reductase from *Mycobacterium marinum*; sfp = maturation factor phosphopantetheinyl transferase from *Bacillus subtilis*; CvFAP = fatty acid photodecarboxylase from *Chlorella variabilis*; ADO = aldehyde deformylating oxygenase from *Clostridium beijerinckii*; Ferr = ferredoxin from *Synechocystis* sp PCC6803 and KdcA = branched-chain keto acid decarboxylase from *Lactococcus lactis*. AlDH enzymes: αKGSDH = α-ketoglutaric semialdehyde dehydrogenase from *Burkholderia ambifaria*; PadA = phenylacetaldehyde dehydrogenase 17 from *E. coli*; or Hpad = 3-hydroxypropionaldehyde dehydrogenase puuC from *E. coli*.

**Figure S2**. Potential amino acid derived biological routes for the production of butane. Pathway for the production of a) butane from isoleucine and d) alternative route to butane from threonine. Enzymes: ilvE = leucine 2-oxoglutarate transaminase from *E. coli*; BCKDHAB = human branched-chain α-keto acid dehydrogenase complex; LeuA = 2-isopropylmalate synthase from *E. coli*; LeuB = 2-isopropylmalate dehydrogenase from *E. coli*; LeuC/LeuD = isopropyl malate isomerase complex from *E. coli*; YciA = acyl-CoA thioester hydrolase from *Haemophilus influenza*; CAR = carboxylic acid reductase from *Mycobacterium marinum*; sfp = maturation factor phosphopantetheinyl transferase from *Bacillus subtilis*; CvFAP = fatty acid photodecarboxylase from *Chlorella variabilis*; ADO = aldehyde deformylating oxygenase from *Clostridium beijerinckii*; Ferr = ferredoxin from *Synechocystis* sp PCC6803; KdcA = branched-chain keto acid decarboxylase from *Lactococcus lactis*; and ilvA = threonine dehydratase from *E. coli*. AlDH enzymes: αKGSDH = α-ketoglutaric semialdehyde dehydrogenase from *Burkholderia ambifaria*; PadA = phenylacetaldehyde dehydrogenase 17 from *E. coli*; or Hpad = 3-hydroxypropionaldehyde dehydrogenase puuC from *E. coli*.

The alternative CoA-dependent route from threonine to butane has an initial ilvE step substituted for threonine dehydratase (ilvA) from *E. coli*. This enzyme catalyses the two-step production of α-ketobutyrate and ammonia from threonine. Two individual chain extension steps (2 x CH_2_) are subsequently performed on α-ketobutyrate by the action of the *E. coli* leuABCD operon to generate 2-ketocaproate (α-keto acid). Valeraldehyde is then formed by the action of BCKDHAB and YciA, or these enzymes can be replaced by a single aldehyde dehydrogenase step. The remaining step(s) are the same as those described for the CoA-dependent routes to form butane. Soluble expression of all the enzymes was seen, except for one of the four LeuABCD genes (Additional File 1: **Fig. S3-S4**). Therefore, the threonine to butane pathway was not investigated further in this study.

**Figure S3.** SDS PAGE analysis of recombinant protein expression in *E. coli*. a) LeuABCD cell extract. Lane 1 = protein ladder; lane 2 = LeuABCD extract. Expected protein masses are listed in **Figure S4** legend. b) Alcohol dehydrogenases αKGSDH (lanes 1-3), PadA (lanes 4-6) and Hpad (lanes 8-10). Protein ladder is shown in lane 7. P = cell pellet slurry; T= total lysate (soluble and insoluble) and S = soluble lysate.

**Figure S4.** Western blot analysis of recombinant protein expression in *E. coli*. a) Lanes: 2 = Hpad; 3 = ilvE; 4 = PadA; 6 = LeuABCD control (no His_6_-tags); 7 = BCKDHAB; 8 = αKGSDH and 9 = ilvA. Protein ladders are located in lanes 1, 5 and 10. Expected protein masses (kDa): Hpad = 56.5; ilvE = 35.2; PadA = 54.7; BCKDH B subunit = 45.6; αKGSDH = 53.9; ilvA = 57.0; LeuA = 57.3; LeuB; 39.5; LeuC = 39.5 and LeuD = 22.5. b) BCKDHAB expressed in three different *E. coli* strains. Strains: lane 2-3 = BL21(DE3); 4 = NiCo21(DE3) and 5 = BL21(DE3) pLysS. The protein ladder is located in lane 1.

**Figure S5.** Effect of *E. coli* strain on hydrocarbon production using construct p*Pr**AFYSCIB. Cultures (10 mL) were grown in LB medium containing 30 µg/ml kanamycin for 4-6 h (OD_600_ ~ 1) at 37 ºC and 180 rpm. Triplicate aliquots (1 mL) each of 3 biological replicate cultures were sealed into glass vials (3 mL) and incubated at 30 ºC for 16-18 h at 200 rpm, illuminated continuously with a blue LED (455 nm or 470 nm). Gaseous hydrocarbon levels were determined by manual headspace injection using an Agilent 490 Micro GC, containing an Al_2_O_3_/KCl column. The errors represent one standard deviation of the data. BL21(DE3) del = BL21(DE3)∆yqhD/∆yjgB double deletion strain.


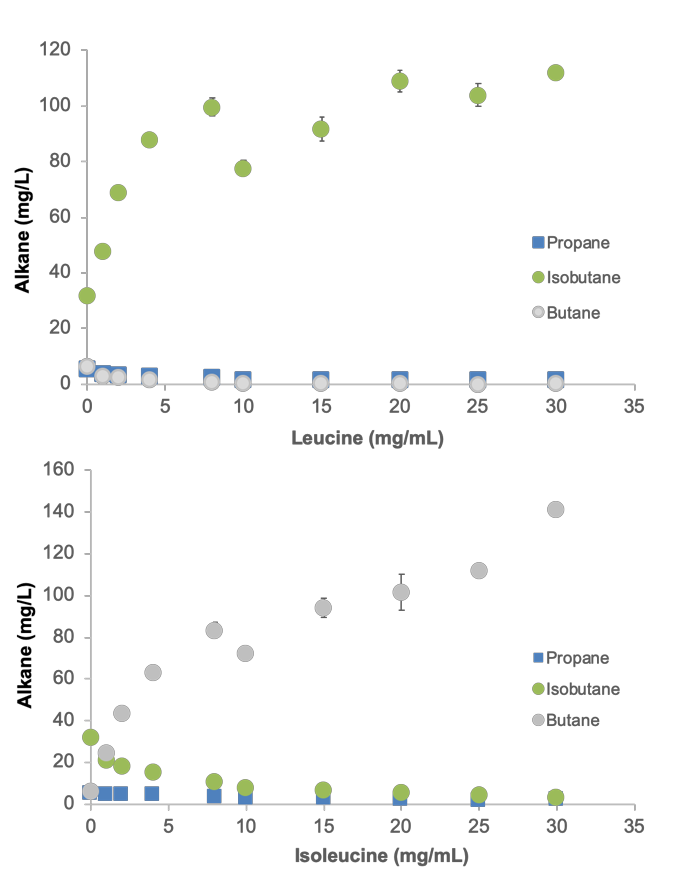


**Figure S6.** Effect of amino acid concentration on hydrocarbon production in *E. coli* expressing construct p*Tr*IH*Tr*KF_G462I_. Cultures (20 mL) were grown in LB medium containing 30 µg/ml kanamycin for 4-6 h (OD_600_ ~ 0.6-0.8) at 37 ºC and 180 rpm. Recombinant protein expression was induced with IPTG (0.1 mM) followed by culture supplementation with amino acids (0-30 mg/mL) after 1 h at 30 °C. Triplicate aliquots (1 mL) each of 3 biological replicate cultures were sealed into glass vials (4 mL) and incubated at 30 ºC for 16-18 h at 200 rpm, illuminated continuously with a blue LED (455 nm or 470 nm). Gaseous hydrocarbon levels were determined by manual headspace injection using an Agilent 490 Micro GC, containing an Al_2_O_3_/KCl column. The errors represent one standard deviation of the data.


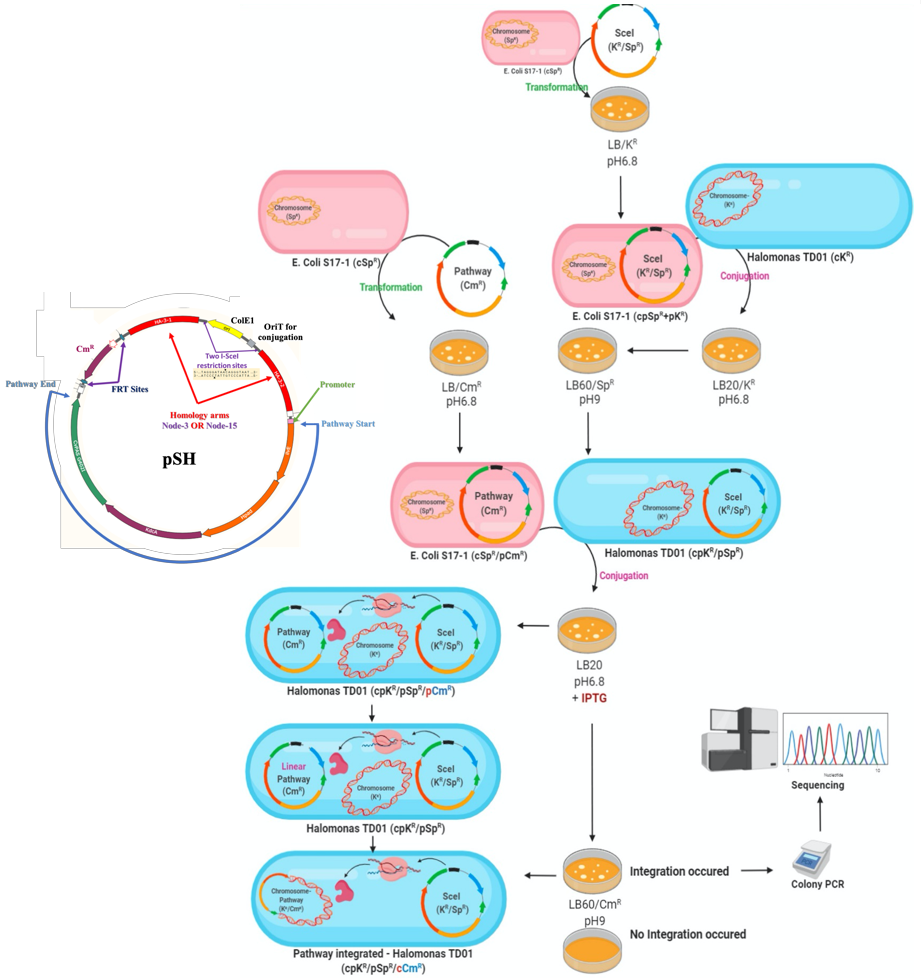


**Figure S7.** Overview of the multi-gene construct chromosomal integration method for *Halomonas*. Created with BioRender.com (from Insightful Science).


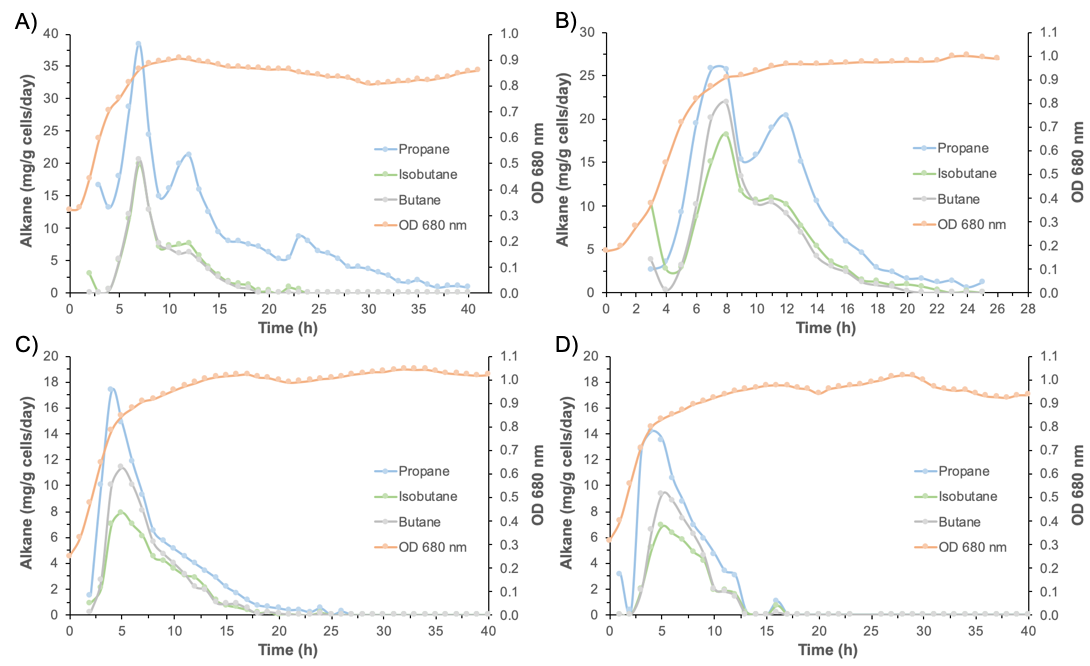


**Figure S8.** Bio-alkane production by *Halomonas* TQ10 in high salt glycerol medium expressing plasmid-borne pH*T7L*IHKF_G462I_. Photobioreactor cultivation (400 mL) was performed with high salt glycerol medium pH 6.8 containing 0.5 mL/L antifoam and 50 μg/mL spectinomycin. Cultivation was performed in batch mode, pre-equilibrated at 30 °C with 60% stirring output with an airflow rate of 1.21 L/min, and ambient room lighting until mid-log phase (4-5 hours). Protein expression was induced by 0.1 mM IPTG, and the culture was maintained for up to 48 h with blue light exposure (1656). Alkane gas production was monitored at 20 min intervals by automated headspace sampling using a Micro GC.


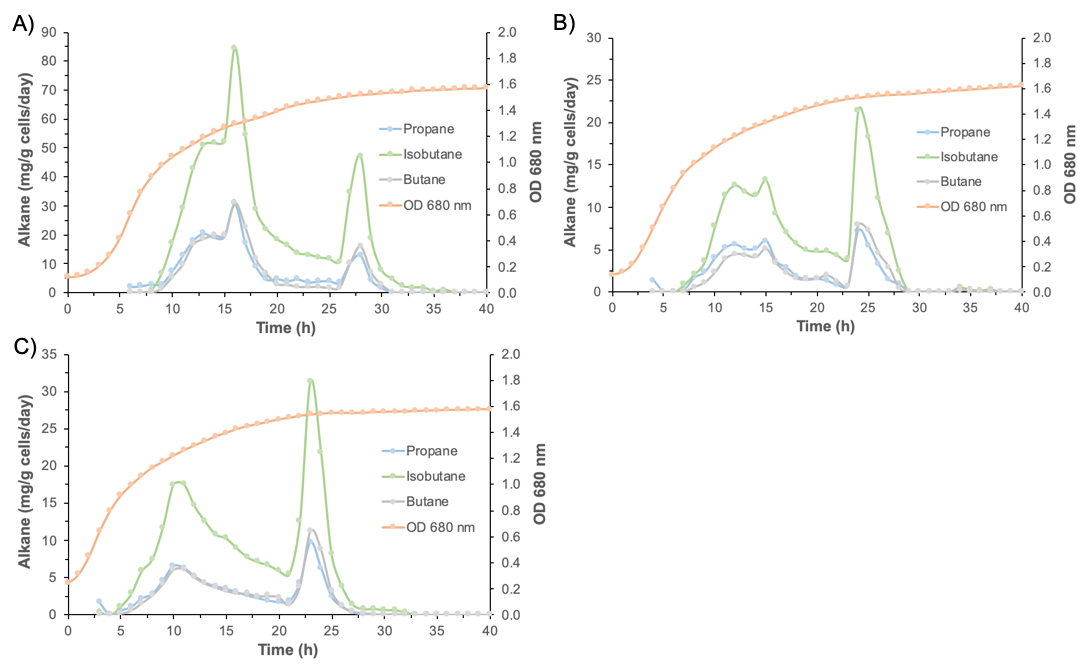


**Figure S9.** Bio-alkane production by *Halomonas* TQ10 in casamino acid supplemented high salt medium expressing plasmid-borne pH*T7L*IHKF_G462I_. Photobioreactor cultivation (400 mL) was performed with LB60Cas medium pH 6.8 containing 0.5 mL/L antifoam and 50 μg/mL spectinomycin. Cultivation was performed in batch mode, pre-equilibrated at 30 °C with 60% stirring output with an airflow rate of 1.21 L/min, and ambient room lighting until mid-log phase (4-5 hours). Protein expression was induced by 0.1 mM IPTG, and the culture was maintained for up to 48 h with blue light exposure (1656). Alkane gas production was monitored at 20 min intervals by automated headspace sampling using a Micro GC.


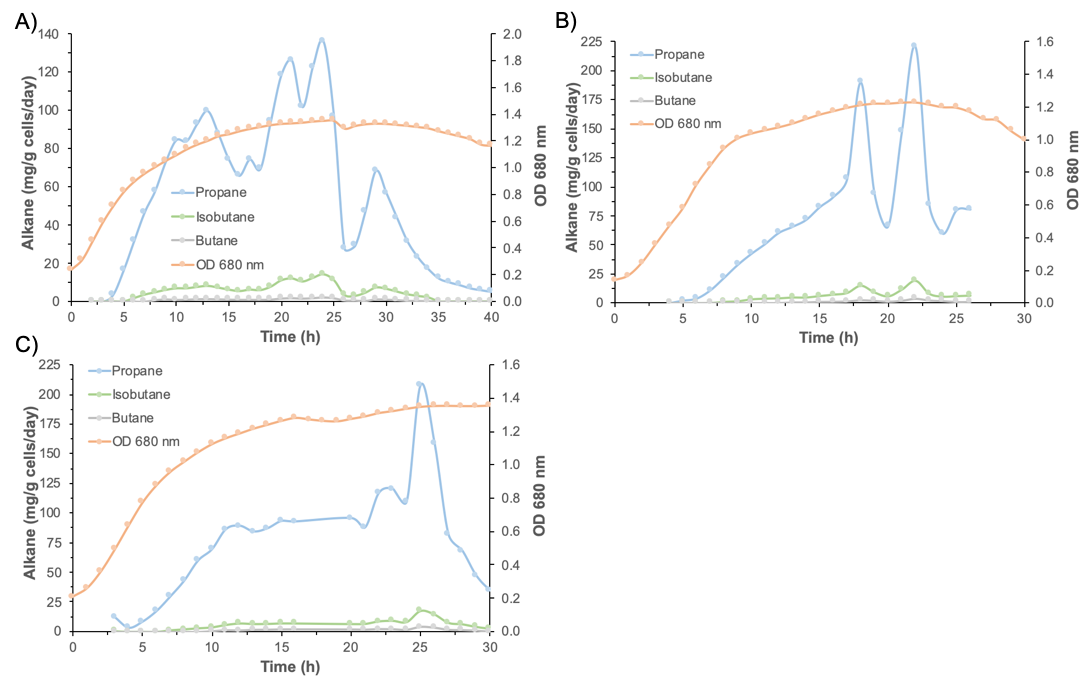


**Figure S10.** Bio-alkane production by *Halomonas* TQ10 in valine supplemented high salt medium expressing plasmid-borne pH*T7L*IHKF_G462I_. Photobioreactor cultivation (400 mL) was performed with LB60Val medium pH 6.8 containing 0.5 mL/L antifoam and 50 μg/mL spectinomycin. Cultivation was performed in batch mode, pre-equilibrated at 30 °C with 60% stirring output with an airflow rate of 1.21 L/min, and ambient room lighting until mid-log phase (4-5 hours). Protein expression was induced by 0.1 mM IPTG, and the culture was maintained for up to 48 h with blue light exposure (1656). Alkane gas production was monitored at 20 min intervals by automated headspace sampling using a Micro GC.


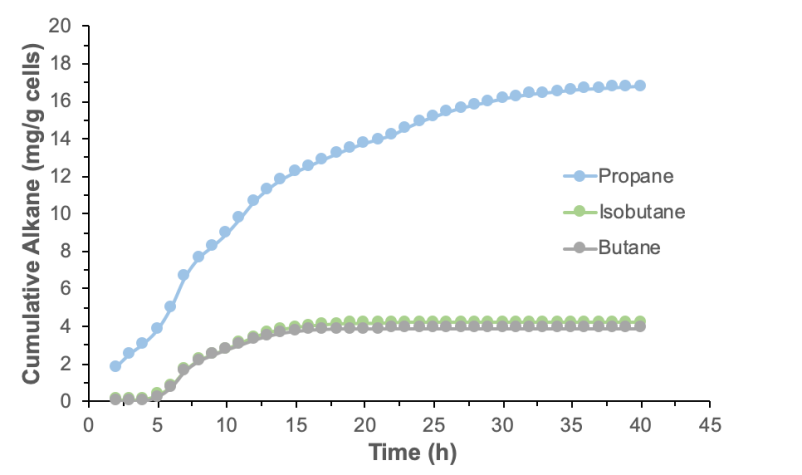


**Figure S11.** Cumulative bio-alkane production by *Halomonas* TQ10 in high salt glycerol medium expressing plasmid-borne pH*T7L*IHKF_G462I_. Photobioreactor cultivation (400 mL) was performed with high salt glycerol medium pH 6.8 containing 0.5 mL/L antifoam and 50 μg/mL spectinomycin. Cultivation was performed in batch mode, pre-equilibrated at 30 °C with 60% stirring output with an airflow rate of 1.21 L/min, and ambient room lighting until mid-log phase (4-5 hours). Protein expression was induced by 0.1 mM IPTG, and the culture was maintained for up to 48 h with blue light exposure (1656). Alkane gas production was monitored at 20 min intervals by automated headspace sampling using a Micro GC.


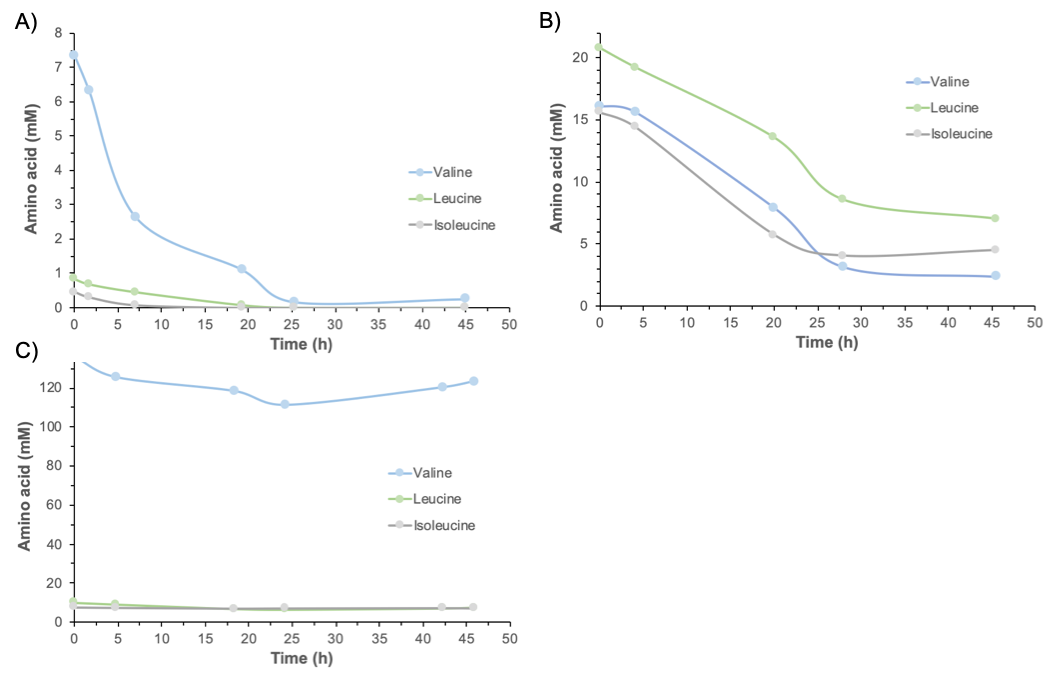


**Figure S12.** Amino acid depletion during fermentation of *Halomonas* TQ10 expressing plasmid-borne pH*T7L*IHKF_G462I_ in three media. Amino acid data is shown for a representative example of cultivation for around 48 h without feeding in A) glycerol, B) LB60Cas and C) LB60Val medium. Methodology of the fermentation runs is described in the legends of Figures S7-S9, respectively. Amino acid concentration determination was performed by manual culture sampling, followed by filtration (0.2 μm) and HPLC analysis. Analysis was performed according to the method of Bartolomeo and Maisano (2006) [1].

**Additional Tables**

| **Table S1.** Effect of *E. coli* strain on alkane production with p*Pr**AFYSCIB construct. | | | | | | |
| --- | --- | --- | --- | --- | --- | --- |
| **Strain** | **Hydrocarbon (μg/L)** | | | **Gas Ratio (%)** | | |
|  | **Propane** | ***iso*-Butane** | ***n*-Butane** | **Propane** | ***iso*-Butane** | ***n*-Butane** |
| BL21(DE3) | 4 ± 1 | 1 ± 0 | 1 ± 0 | 79 | 15 | 6 |
| NiCo2(DE3) | 17 ± 3 | 6 ± 2 | 3 ± 0 | 71 | 20 | 9 |
| BL21(DE3)∆∆ | 110 ± 7 | 27 ± 5 | 12 ± 2 | 77 | 15 | 8 |
| Cultures (10 mL) were grown in LB medium containing 30 µg/ml kanamycin for 4-6 h (OD_600_ ~ 1) at 37 ºC and 180 rpm. Triplicate aliquots (1 mL) each of 3 biological replicate cultures were sealed into glass vials (3 mL) and incubated at 30 ºC for 16-18 h at 200 rpm, illuminated continuously with a blue LED (455 nm or 470 nm). Gaseous hydrocarbon levels were determined by manual headspace injection using an Agilent 490 Micro GC, containing an Al_2_O_3_/KCl column. nd = none detected. BL21(DE3)∆∆ = BL21(DE3) ∆yqhD/∆yjgB | | | | | | |

| **Table S2.** Alkane production in *E. coli* expressing recombinant ADO- and CvFAP-dependent pathways from amino acids. | | | | | | |
| --- | --- | --- | --- | --- | --- | --- |
| **Construct** | **Hydrocarbon (mg/L)** | | | **Gas Ratio (%)** | | |
|  | **Propane** | ***iso*-Butane** | ***n*-Butane** | **Propane** | ***iso*-Butane** | ***n*-Butane** |
| *CoA-dependent route with ADO_A134F_* | | | | | | |
| p*Pr**AFYSCIB | 0.017 ± 0.002 | 0.002 ± 0.001 | nd | 89 | 11 | - |
| p*Pr**AFYSC*Pr*IB | nd | nd | nd | - | - | - |
| p*Tr*AFYSCIB | 0.36 ± 0.01 | 0.017 ± 0.005 | 0.021 ± 0.006 | 90 | 4 | 5 |
| p*Tr*AFYSC*Tr*IB | 0.010 ± 0.003 | nd | nd | 100 | - | - |
| p*Tr*Δ*L*AFYSCIB | 0.956 ± 0.008 | 0.030 ± 0.001 | 0.012 ± 0.001 | 96 | 3 | 1 |
| *CoA-dependent route with CvFAP_G462V_* | | | | | | |
| p*Tr*F_G462V_YIB | 0.98 ± 0.06 | 0.09 ± 0.02 | 0.04 ± 0.01 | 88 | 8 | 4 |
| p*Pr*F_G462V_YIB | nd | nd | nd | - | - | - |
| *CoA-dependent route with ADO_A134F_ and CvFAP_G462V_* | | | | | | |
| p*Tr*AFYSCIB with p*Tr*CvFAP_G462V_ | 0.26 ± 0.04 | 0.030 ± 0.004 | 0.030 ± 0.001 | 81 | 9.5 | 9.5 |
| p*Pr*AFYSCIB with p*Tr*CvFAP_G462V_ | 0.15 ± 0.02 | 0.03 ± 0.01 | nd | 83 | 17 | - |
| *KdcA-dependent route with CvFAP_G462V_ or CvFAP_G462I_* | | | | | | |
| p*Tr*IαK*Tr*KF_G462V_ | 4.24 ± 0.11 | 14.71 ± 0.41 | 2.18 ± 0.08 | 20 | 70 | 10 |
| p*Tr*IP*Tr*KF_G462V_ | 4.26 ± 0.25 | 13.77 ± 0.74 | 2.72 ± 0.13 | 21 | 66 | 13 |
| p*Tr*IH*Tr*KF_G462V_ | 5.07 ± 0.14 | 15.99 ± 0.64 | 3.20 ± 0.19 | 21 | 66 | 13 |
| p*Tr*IαK*Tr*KF_G462I_ | 5.55 ± 0.36 | 30.40 ± 2.10 | 5.63 ± 0.48 | 13 | 73 | 14 |
| p*Tr*IP*Tr*KF_G462I_ | 6.06 ± 0.54 | 28.77 ± 2.12 | 7.18 ± 0.42 | 14 | 68 | 17 |
| p*Tr*IH*Tr*KF_G462I_ | 7.77 ± 0.54 | 38.55 ± 2.54 | 9.58 ± 0.55 | 14 | 69 | 17 |
| Cultures (10 mL) were grown in LB medium containing the required antibiotics (**Table S5**) for 4-6 h (OD_600_ ~ 1) at 37 ºC and 180 rpm. Protein induction (100 μM IPTG) was performed for *trc*-containing constructs, and triplicate aliquots (1 mL) each of 3 biological replicate cultures were sealed into glass vials (3 mL) and incubated at 30 ºC for 16-18 h at 200 rpm, illuminated continuously with a blue LED (455 nm or 470 nm). Gaseous hydrocarbon levels were determined by manual headspace injection using an Agilent 490 Micro GC, containing an Al_2_O_3_/KCl column. nd = none detected. | | | | | | |

| **Table S3.** Gaseous bio-alkane production in *E. coli* BL21(DE3)ΔΔ expressing constructs in two plasmid backbones and one or two promoters. | | | | | | |
| --- | --- | --- | --- | --- | --- | --- |
| **Construct** | **Hydrocarbon (mg/L)** | | | **Gas Ratio (%)** | | |
|  | **Propane** | ***iso*-Butane** | ***n*-Butane** | **Propane** | ***iso*-Butane** | ***n*-Butane** |
| *Plasmid backbone – pBbE1k* | | | | | | |
| p*Tr*IH*Tr*KF_G462I_ | 5.80 ± 0.11  6.18 ± 0.38^a^ | 32.15 ± 0.48  12.31 ± 0.59^a^ | 6.65 ± 0.02  3.12 ± 0.14^a^ | 13  29^a^ | 72  57^a^ | 15  14^a^ |
| p*Tr*IHKF_G462I_ | 5.49 ± 0.40  5.22 ± 0.24^a^ | 29.61 ± 2.17  10.60 ± 0.44^a^ | 6.19 ± 0.57  2.63 ± 0.12^a^ | 13  28^a^ | 72  57^a^ | 15  14^a^ |
| p*c*Δ*L*IHKF_G462I_ | 2.08 ± 0.03 | 11.10 ± 0.08 | 2.07 ± 0.01 | 14 | 73 | 14 |
| p*cTr*Δ*L*IHKF_G462I_ | 2.02 ± 0.02 | 8.61 ± 0.18 | 1.65 ± 0.03 | 16 | 70 | 13 |
| *Plasmid backbone – pBbE1a* | | | | | | |
| p*Tr*IH*Tr*KF_G462I_E1a | 3.24 ± 0.24 | 16.89 ± 1.29 | 3.33 ± 0.24 | 14 | 72 | 14 |
| p*Tr*IHKF_G462I_E1a | 2.91 ± 0.14 | 16.16 ± 0.65 | 2.62 ± 0.09 | 13 | 75 | 12 |
| Cultures (10 mL) were grown in LB, or ^a^phosphate buffered TB medium, containing 30 μg/mL kanamycin for 4-6 h (OD_600_ ~ 1) at 37 ºC and 180 rpm. Protein induction (100 μM IPTG) was performed, and triplicate aliquots (1 mL) each of 3 biological replicate cultures were sealed into glass vials (3 mL) and incubated at 30 ºC for 16-18 h at 200 rpm, illuminated continuously with a blue LED (455 nm or 470 nm). Gaseous hydrocarbon levels were determined by manual headspace injection using an Agilent 490 Micro GC, containing an Al_2_O_3_/KCl column. Constitutive promoters: *c*Δ*L* = *lacI* promoter minus lacI^q^; *cTr*Δ*L* = *trc* promoter minus lacI^q^. | | | | | | |

| **Table S4.** Gaseous bio-alkane production in *E. coli* expressing p*Tr*IH*Tr*KF_G462I_ construct. | | | | | | |
| --- | --- | --- | --- | --- | --- | --- |
| **Supplemental**  **Amino acid (mg/mL)**^1^ | **Hydrocarbon (mg/L)** | | | **Gas Ratio (%)** | | |
|  | **Propane** | ***iso*-Butane** | ***n*-Butane** | **Propane** | ***iso*-Butane** | ***n*-Butane** |
| 0 | 5.80 ± 0.11 | 32.15 ± 0.48 | 6.65 ± 0.02 | 13 | 72 | 15 |
| *Leucine supplementation* | | | | | | |
| 1 | 3.93 ± 0.08 | 47.86 ± 1.41 | 3.11 ± 0.10 | 7 | 87 | 6 |
| 2 | 3.51 ± 0.05 | 69.27 ± 0.70 | 2.49 ± 0.06 | 5 | 92 | 3 |
| 4 | 3.31 ± 0.11 | 88.13 ± 1.52 | 1.91 ± 0.04 | 4 | 94 | 2 |
| 8 | 2.65 ± 0.12 | 99.79 ± 3.18 | 1.15 ± 0.04 | 3 | 96 | 1 |
| 10 | 2.00 ± 0.33 | 77.85 ± 2.72 | 0.55 ± 0.03 | 2 | 97 | 1 |
| 15 | 1.85 ± 0.07 | 91.79 ± 4.33 | 0.41 ± 0.02 | 2 | 98 | <1 |
| 20 | 1.93 ± 0.03 | 109.04 ± 3.89 | 0.34 ± 0.01 | 2 | 98 | <1 |
| 25 | 1.86 ± 0.08 | 103.97 ± 3.92 | 0.27 ± 0.01 | 2 | 98 | <1 |
| 30 | 1.86 ± 0.03 | 112.10 ± 0.59 | 0.32 ± 0.00 | 2 | 98 | <1 |
| *Isoleucine supplementation* | | | | | | |
| 1 | 4.76 ± 0.06 | 21.22 ± 0.33 | 25.08 ± 0.28 | 9 | 42 | 49 |
| 2 | 4.91 ± 0.14 | 18.75 ± 0.39 | 44.05 ± 1.09 | 7 | 28 | 65 |
| 4 | 4.80 ± 0.12 | 15.63 ± 0.29 | 63.46 ± 0.99 | 6 | 18 | 76 |
| 8 | 4.12 ± 0.22 | 11.32 ± 0.48 | 83.50 ± 3.57 | 4 | 12 | 84 |
| 10 | 3.04 ± 0.05 | 8.32 ± 1.00 | 72.68 ± 1.47 | 4 | 10 | 86 |
| 15 | 3.02 ± 0.11 | 6.79 ± 0.25 | 94.17 ± 4.46 | 3 | 7 | 90 |
| 20 | 2.61 ± 0.15 | 5.59 ± 0.35 | 101.62 ± 8.42 | 2 | 5 | 93 |
| 25 | 2.37 ± 0.07 | 4.64 ± 0.08 | 112.09 ± 1.92 | 2 | 4 | 94 |
| 30 | 2.43 ± 0.05 | 3.56 ± 0.06 | 141.59 ± 2.98 | 2 | 2 | 96 |
| Cultures (10 mL) were grown in LB containing 30 μg/mL kanamycin for 4-6 h (OD_600_ ~ 1) at 37 ºC and 180 rpm. Protein induction (100 μM IPTG) was performed followed by amino acid addition (0-30 mg/mL) and triplicate aliquots (1 mL) each of 3 biological replicate cultures were sealed into glass vials (3 mL) and incubated at 30 ºC for 16-18 h at 200 rpm, illuminated continuously with a blue LED (455 nm or 470 nm). Gaseous hydrocarbon levels were determined by manual headspace injection using an Agilent 490 Micro GC, containing an Al_2_O_3_/KCl column. ^1^Data for valine supplementation was previously published [2], so has not been recorded in this Table. In the absence of amino acid supplementation, the approximate concentrations of valine, leucine and isoleucine in Luria broth are reported to be 7, 5.4 and 8.8 mM, respectively [3]. | | | | | | |

| **Table S5.** Gaseous bio-alkane production in *Halomonas* TQ10 containing either plasmid-borne or chromosomally integrated KdcA-dependent constructs. | | | | | | |
| --- | --- | --- | --- | --- | --- | --- |
| **Construct** | **Hydrocarbon (mg/L)** | | | **Gas Ratio (%)** | | |
|  | **Propane** | ***iso*-Butane** | ***n*-Butane** | **Propane** | ***iso*-Butane** | ***n*-Butane** |
| *Plasmid constructs* | | | | | | |
| pH*T7L*IHKF_G462I_^a^ | 3.78 ± 0.26  7.99 ± 1.07^b^ | 9.32 ± 0.63  0.65 ± 0.08^b^ | 2.94 ± 0.22  0.11 ± 0.01^b^ | 24  91 | 58  7 | 18  1 |
| pH*cT7L∆L*IHKF_G462I_ | 0.86 ± 0.14  2.32 ± 0.32^b^ | 2.08 ± 0.39  0.14 ± 0.00^b^ | 0.56 ± 0.10  0.01 ± 0.00^b^ | 25  94 | 59  6 | 16  0 |
| pH*c∆L*IHKF_G462I_ | 1.81 ± 0.06  3.95 ± 0.16^b^ | 1.91 ± 0.10  0.37 ± 0.03^b^ | 0.73 ± 0.03  0.02 ± 0.00^b^ | 41  91 | 43  9 | 16  0 |
| pH*c69*IHKF_G462I_^a^ | 0.97 ± 0.01 | 1.18 ± 0.02 | 0.41 ± 0.01 | 38 | 46 | 16 |
| pH*c59*IHKF_G462I_ | 0.15 ± 0.00 | 0.35 ± 0.02 | 0.12 ± 0.00 | 24 | 57 | 19 |
| pH*c102*IHKF_G462I_ | 0.04 ± 0.00 | 0.04 ± 0.00 | 0.01 ± 0.00 | 45 | 44 | 11 |
| *Chromosomal integrated constructs* | | | | | | |
| N3*T7L*IHKF_G462I_^a^ | 0.71 ± 0.04  2.70 ± 0.10^b^ | 1.39 ± 0.09  0.29 ± 0.01^b^ | 0.66 ± 0.05  0.04 ± 0.00^b^ | 26  89 | 50  10 | 24  1 |
| N3*cT7L∆L*IHKF_G462I_ | 0.25 ± 0.00  1.72 ± 0.16^b^ | 0.43 ± 0.00  0.15 ± 0.02^b^ | 0.20 ± 0.00  0.02 ± 0.00^b^ | 28  91 | 49  8 | 23  1 |
| N3*c∆L*IHKF_G462I_ | 0.68 ± 0.01  1.84 ± 0.28^b^ | 1.19 ± 0.01  0.12 ± 0.05^b^ | 0.58 ± 0.01  0.02 ± 0.02^b^ | 28  93 | 49  8 | 24  1 |
| N3*c69*IHKF_G462I_^a^ | 0.29 ± 0.01 | 0.47 ± 0.02 | 0.23 ± 0.01 | 29 | 47 | 24 |
| N15*T7L*IHKF_G462I_ | 0.17 ± 0.01 | 0.24 ± 0.03 | 0.14 ± 0.02 | 31 | 44 | 25 |
| N15*cT7L∆L*IHKF_G462I_ | 0.15 ± 0.00 | 0.19 ± 0.00 | 0.11 ± 0.00 | 27 | 35 | 20 |
| N15*c∆L*IHKF_G462I_ | 0.16 ± 0.00 | 0.25 ± 0.01 | 0.13 ± 0.00 | 30 | 45 | 23 |
| N15*c69*IHKF_G462I_ | 0.07 ± 0.00 | 0.10 ± 0.00 | 0.05 ± 0.00 | 13 | 17 | 9 |
| ^a^Data obtained from previously published studies [2]. ^b^Cultures contained supplemental valine (30 mg/mL). Cultures were grown in LB medium pH 9.0 with/without supplemental valine (30 mg/mL) containing 60 g/L NaCl and spectinomycin (50 μg/mL) for 5 h at 37 °C and 180 rpm. Recombinant protein expression was induced with IPTG (0.1 mM; OD_600nm_ ~ 1.6), and triplicate aliquots (1 mL) of cultures were sealed into 4 mL glass vials and incubated at 30 °C for 16-18 h at 200 rpm, illuminated with a blue LED panel. Headspace gas was analysed for hydrocarbon content using a Micro GC. Gaseous hydrocarbon levels were determined by manual headspace injection using an Agilent 490 Micro GC, containing an Al_2_O_3_/KCl column. Constitutive promoters: *c*Δ*L* = *lacI* promoter minus lacI^q^; *cTr*Δ*L* = *trc* promoter minus lacI^q^. | | | | | | |

| Table S6. Inducible plasmids used in this study. | | |  |
| --- | --- | --- | --- |
| Construct | Promoter-Gene(s) | Plasmid | Antibiotic |
| *Expression in E. coli* | | | |
| pADO_A134F_ | *T7*-ADO_A134F_ | pCDFDuet-1[4] | Spectinomycin |
| pFerr | *T7*-Ferr | pRSF-Duet1[4] | Kanamycin |
| pTPC7 | *T7*-YciA-sfp-CAR | pETDuetT-1[4] | Ampicillin |
| pCvFAP_G462V_[5] | *T7*-CvFAP_G462V_ | pETM11 | Kanamycin |
| pilvE | *T7*-ilvE | pET21b(+) | Ampicillin |
| pBCKDHAB | *T7*-BCKDHAB | pET21b(+) | Ampicillin |
| paKGSDH | *T7*-αKGSDH | pETM11 | Kanamycin |
| pPadA | *T7*-PadA | pET21b(+) | Ampicillin |
| pHpad | *T7*-Hpad | pETM11 | Kanamycin |
| pKdcA | kdcA | pMA-T^a^ | Ampicillin |
| pilvA | *T7*-ilvA | pET21b(+) | Ampicillin |
| pLeuABCD | *T7-LeuABCD* | pET21b(+) | Ampicillin |
| p*Tet*ADO_A134F_Ferr | *Tet*-ADO_A134F_-Ferr | pBbE2k | Kanamycin |
| pAFYSC | *T7*-ADO_A134F_-Ferr-YciA-sfp-CAR | pETDuetT-1 | Ampicillin |
| p*Tr*AFYSCIB | *trc*-ADO_A134F_-Ferr-YciA-sfp-CAR-ilvE-BCKDHAB | pBbE1k | Kanamycin |
| p*Tr*AFYSC*Tr*IB | *trc*-ADO_A134F_-Ferr-YciA-sfp-CAR-*trc*-ilvE-BCKDHAB | pBbE1k | Kanamycin |
| p*Tr*CvFAP_G462V_ | *trc*-CvFAP_G462V_ | pBbA1c | Chloramphenicol |
| p*Tr*F_G462V_YIB | *trc*-CvFAP_G462V_-YciA-ilvE-BCKDHAB | pBbE1k | Kanamycin |
| p*Tr*IaK*Tr*KF_G462V_ | *trc*-ilvE-αKGSDH-*trc*-kdcA-CvFAP_G462V_ | pBbE1k | Kanamycin |
| p*Tr*IP*Tr*KF_G462V_ | *trc*-ilvE-PadA-*trc*-kdcA-CvFAP_G462V_ | pBbE1k | Kanamycin |
| p*Tr*IH*Tr*KF_G462V_ | *trc*-ilvE-Hpad-*trc*-kdcA-CvFAP_G462V_ | pBbE1k | Kanamycin |
| p*Tr*IaK*Tr*KF_G462I_ | *trc*-ilvE-αKGSDH-*trc*-kdcA-CvFAP_G462I_ | pBbE1k | Kanamycin |
| p*Tr*IP*Tr*KF_G462I_ | *trc*-ilvE-PadA-*trc*-kdcA-CvFAP_G462I_ | pBbE1k | Kanamycin |
| p*Tr*IH*Tr*KF_G462I_ | *trc*-ilvE-Hpad-*trc*-kdcA-CvFAP_G462I_ | pBbE1k | Kanamycin |
| p*Tr*IHKF_G462I_ | *trc*-ilvE-Hpad-kdcA-CvFAP_G462I_ | pBbE1k | Kanamycin |
| p*Tr*IH*Tr*KF_G462I_E1a | *trc*-ilvE-Hpad-*trc*-kdcA-CvFAP_G462I_ | pBbE1a | Kanamycin |
| p*Tr*IHKF_G462I_E1a | *trc*-ilvE-Hpad-kdcA-CvFAP_G462I_ | pBbE1a | Kanamycin |
| *Expression in Halomonas* | | | |
| pH*T7L*IHKF_G462I_ | *Mmp1*-ilvE-Hpad-kdcA-CvFAP_G462I_ | pHal2 | Spectinomycin |
| pH*T7L*-RFP | *Mmp1*-RFP | pHal2 | Spectinomycin |
| pSH-N3^b^ | Contains homology arms for Node 3 | pSEVA241 | Chloramphenicol |
| pSH-N15^b^ | Contains homology arms for Node 15 | pSEVA241 | Chloramphenicol |
| pSBR1Ks-i-SceI | *pTrc*-SceI | pHal2 | Spectinomycin |
| ^a^Cloning vector from GeneArt. ^b^Based on the CRISPR/Cas9 editing *Halomonas* genome donor DNA pSEVA241 plasmid of Quin, Q. *et al* [6], except the gRNA and antibiotic resistances were removed, and the insert contained the target DNA with a pKIKO-derived chloramphenicol resistance gene flanked by FRT sequences [7]. Promoters: *T7* = T7lac IPTG-inducible promoter; *trc* = IPTG-inducible hybrid of the *lac* and *trc* promoters; proD and R0011 = constitutive promoters [8] and *Tet* = tetracycline inducible promoter [9]. p*Pr** = proD promoter with the residual LacI^q^ removed. pΔ*L* = p*Tr* promoter minus *trc* and *lacI*. Promoters are shown in italics and amino acid changes are shown as a subscript. *T7L* = *Halomonas*-compatible T7-like IPTG-inducible MMP1 promoter [10]. The pBb series of vectors were obtained from Addgene [9]. *Halomonas* vector pHal2 is described in [2]. SceI – gene coding for endonuclease SceI. | | | |

| Table S7. Constitutive plasmids used in this study. | | |  |
| --- | --- | --- | --- |
| Construct | Promoter-Gene(s) | Plasmid | Antibiotic |
| *Expression in E. coli* | | | |
| p*Pr*ADO_A134F_ | *proD-*ADO_A134F_ | pBbEproDK^a^ | Kanamycin |
| pYSCAP | *R0011*-YciA-sfp-CAR-*proD*-ADO_A134F_-Ferr | pG9^c^ | Ampicillin |
| p*Pr**AFYSC | *proD**-ADO_A134F_-Ferr-YciA-sfp-CAR | pBbEproDK^a^ | Kanamycin |
| p*Pr*AFYSC | *proD*-ADO_A134F_-Ferr-YciA-sfp-CAR | pBbEproDK^a,b^ | Kanamycin |
| p*Pr**IB | *proD**-ilvE-BCKDHAB | pBbEproDK^a^ | Kanamycin |
| p*Pr**AFYSCIB | *proD**-ADO_A134F_-Ferr-YciA-sfp-CAR-ilvE-BCKDHAB | pBbEproDK^a^ | Kanamycin |
| p*Pr*AFYSC*Pr* | *proD*-ADO_A134F_-Ferr-YciA-sfp-CAR-*proD* | pBbEproDK^a,b^ | Kanamycin |
| p*Pr**AFYSC*Pr*IB | *proD**-ADO_A134F_-Ferr-YciA-sfp-CAR-*proD*-ilvE-BCKDHAB | pBbEproDK^a^ | Kanamycin |
| p*cTr*Δ*L*AFYSCIB | *trc(*ΔLacI)-ADO_A134F_-Ferr-YciA-sfp-CAR-ilvE-BCKDHAB | pBbE1k[9] | Kanamycin |
| p*Pr*F_G462V_YIB | *proD*-CvFAP_G462V_-YciA-ilvE-BCKDHAB | pBbEproDK^a^ | Kanamycin |
| p*c*Δ*L*IH*Tr*KF_G462I_ | *trc(*Δ*trc*ΔLacI)-ilvE-Hpad-*trc*-kdcA-CvFAP_G462I_ | pBbE1k[9] | Kanamycin |
| p*cTr*Δ*L*IHKF_G462I_ | *trc(*ΔLacI)-ilvE-Hpad-kdcA-CvFAP_G462I_ | pBbE1k[9] | Kanamycin |
| p*cΔL*IHKF_G462I_ | *trc(*Δ*trc*ΔLacI)-ilvE-Hpad-kdcA-CvFAP_G462I_ | pBbE1k[9] | Kanamycin |
| p*cTrΔL*IHKF_G462I_ | *trc(*ΔLacI)-ilvE-Hpad-kdcA-CvFAP_G462I_ | pBbE1k[9] | Kanamycin |
| *Expression in Halomonas* | | | |
| pH*cT7LΔL*IHKF_G462I_ | *T7LΔL*-ilvE-Hpad-kdcA-CvFAP_G462I_ | pHal2 | Spectinomycin |
| pH*cΔL*IHKF_G462I_ | *trc(*Δ*trc*ΔLacI)-ilvE-Hpad-kdcA-CvFAP_G462I_ | pHal2 | Spectinomycin |
| pH*c102*IHKF_G462I_ | *Porin102*-ilvE-Hpad-kdcA-CvFAP_G462I_ | pHal2 | Spectinomycin |
| pH*c69*IHKF_G462I_ | *Porin69*-ilvE-Hpad-kdcA-CvFAP_G462I_ | pHal2 | Spectinomycin |
| pH*c59*IHKF_G462I_ | *Porin59*-ilvE-Hpad-kdcA-CvFAP_G462I_ | pHal2 | Spectinomycin |
| pH*c102*-RFP | *Porin102*-RFP | pHal2 | Spectinomycin |
| pH*c69*-RFP | *Porin69*-RFP | pHal2 | Spectinomycin |
| pH*c59*-RFP | *Porin59*-RFP | pHal2 | Spectinomycin |
| ^a^Modified pBbE7k vector, where the T7 promoter was substituted for the constitutive promoter proD, and ^b^further modified to remove residual LacI^q^. Promoters: proD and R0011 = constitutive promoters [8]; p*Pr** = proD promoter with the residual LacI^q^ removed. pΔ*L* = p*Tr* promoter minus *trc* and *lacI*. Promoters are shown in italics and amino acid changes are shown as a subscript. *T7L* = *Halomonas*-compatible T7-like IPTG-inducible promoter [10]. *Halomonas* vector pHal2 is described in [2]. | | | |

| Table S8. *Halomonas* chromosomally-integrated constructs used in this study. | | |  |
| --- | --- | --- | --- |
| Construct | Promoter-Gene(s) | Node (loci) | Antibiotic |
| N3*T7L*IHKF_G462I_ | *Mmp1*-ilvE-Hpad-kdcA-CvFAP_G462I_ | 3 | Chloramphenicol |
| N15*T7L*IHKF_G462I_ | *Mmp1*-ilvE-Hpad-kdcA-CvFAP_G462I_ | 15 | Chloramphenicol |
| N3*cT7LΔL*IHKF_G462I_ | *Mmp1ΔL*-ilvE-Hpad-kdcA-CvFAP_G462I_ | 3 | Chloramphenicol |
| N15*cT7LΔL*IHKF_G462I_ | *Mmp1ΔL*-ilvE-Hpad-kdcA-CvFAP_G462I_ | 15 | Chloramphenicol |
| N3*cΔL*IHKF_G462I_ | *ΔL*-ilvE-Hpad-kdcA-CvFAP_G462I_ | 3 | Chloramphenicol |
| N15*cΔL*IHKF_G462I_ | *ΔL*-ilvE-Hpad-kdcA-CvFAP_G462I_ | 15 | Chloramphenicol |
| N3*c69*IHKF_G462I_ | *pPorin69-*ilvE-Hpad-kdcA-CvFAP_G462I_ | 3 | Chloramphenicol |
| N15*c69*IHKF_G462I_ | *pPorin69-*ilvE-Hpad-kdcA-CvFAP_G462I_ | 15 | Chloramphenicol |
| Promoters: *T7L* = *Halomonas*-compatible T7-like IPTG-inducible promoter [10]; *ΔL* = lacI^q^ eliminated from the MmP1 promoter. Promoters are shown in italics and amino acid changes are shown as a subscript. | | | |

| **Table S9.** Oligonucleotide sequences for the assembly of ADO-containing CoA-dependent DNA constructs. | | |
| --- | --- | --- |
| **Stage** | **Oligonucleotide sequence 5’ to 3’** | **Template DNA** |
| *Construction of the constitutive promoter* p*Pr*ADO_A134F_ *by In-Fusion cloning* | | |
| Vector opening  *proD*-ADO PCR | TCCGAGTAAGGATCTCCAGGC  ATTTCGCGGGATCGAGATCG  TCGATCCCGCGAAATGAATTC*CACAGCTAACACCACGTCGTCCCT*  AGATCCTTACTCGGATCCTTAGCTGACTAAGGCTGCTGCCGC | pBbE7k-RFP  pYSCAP |
| *Assembly of* p*Tet*ADO_A134F_Ferr *by In-Fusion cloning* | | |
| Vector opening  ADO PCR  Ferr PCR | GGATCCAAACTCGAGTAAGG  CTTCTTAAAAGATCTTTTGAATTC  AGATCTTTTAAGAAGCCTGTAGAAATAATTTTGTTTAACTTTAATAAGG  ATTTAGCTGTCCTCCTTAAGAAACCAGGGCCGCTGC  GGAGGACAGCTAAATGGCATCCTATACCGTTAAATTGATCACC  CTCGAGTTTGGATCCTTAGTAGAGGTCTTCTTCTTTGTGGGTTTC | pBbE2k-RFP  pADO_A134F_  pFerr |
| *Assembly of* pAFYSC *by In-Fusion cloning* | | |
| Vector opening  ADO-Ferr PCR | AGGAGGAAACATATGTCTGCTAACTTTACTGATAAAAACG  GGTATATCTCCTTCTTAAAGTTAAACAAAATTATTTCTAGAGG  AGAAGGAGATATACCATGGGCAGCAGCC  CATATGTTTCCTCCTTTAGTAGAGGTCTTCTTCTTTGTGGGTTTC | pTPC7  p*Tet*ADO_A134F_Ferr |
| *Assembly of* p*Pr**AFYSC *by In-Fusion cloning* | | |
| Vector opening  AFYSC PCR | TAAGGATCCGAGTAAGGATCTCCAGGCATC  AAAGTTAAACAAAATTATTTGTAGAGGGAAACCGTTGTCG  ATTTTGTTTAACTTTTTTGTTTAACTTTAAGAAGGAGATATACCATG  TTACTCGGATCCTTACAGCAGGCCCAGCAGG | p*Pr*ADO_A134F_  pAFYSC |
| *Elimination of LacI^q^ to generate* p*Pr*AFYSC *by In-Fusion cloning* | | |
| Vector opening | GCAATTCCGACGTCCCGGGATCTCGACGCTCTCCCT  GGACGTCGGAATTGCCAGCTGG | p*Pr*AFYSC |
| *Assembly of* p*Pr**IB *by In-Fusion cloning* | | |
| ilvE PCR  BCKDHAB PCR  OEP  Vector opening | ATTTTGTTTAACTTTTAAGAAGGAGATATACATATGACCACCAAAAAAG  CCTGAATTCGGATCCTCAGTGGTGGTGGTGGTGGTGC  GGATCCGAATTCAGGAGGTAAAAAAATGAGCAGCCTGGATGATAAACCGC  GATCCTTACTCGGATTCAGTGGTGGTGGTGGTGGTGCT  ATTTTGTTTAACTTTTAAGAAGGAGATATACATATGACCACCAAAAAAG  GATCCTTACTCGGATTCAGTGGTGGTGGTGGTGGTGCT  ATCCGAGTAAGGATCTCCAGGCATC  AAGTCATAGCATTTACAGCAGGCCCAGCAGGCG | pilvE  pBCKDHAB  pBCKDHAB & pilvE  p*Pr*AFYSC |
| *Assembly of* p*Pr**AFYSCIB *by In-Fusion cloning* | | |
| Vector opening  Insert IB PCR | ATCCGAGTAAGGATCTCCAGGCATC  AAGTCATAGCATTTACAGCAGGCCCAGCAGGCG  TAAATGCTATGACTTATTTTGTTTAACTTTTAAGAAGGAGATAT  AGATCCTTACTCGGATTCAGTGG | p*Pr*Δ*L*AFYSC  p*Pr*IB |
| *Addition of a second proD promoter to generate* p*Pr*AFYSC*Pr* | | |
| Vector opening  Promoter PCR | ATCCGAGTAAGGATCTCCAGGCATC  AAGTCATAGCATTTACAGCAGGCCCAGCAGGCG  TAAATGCTATGACTTCACAGCTAACACCACGTCGTCCCT  GATCCTTACTCGGATAAAGTTAAACAAAATTATTTGTAGAGGGAAACCGTTG | p*Pr*Δ*L*AFYSC  p*Pr*AFYSC |
| *Assembly of* p*Pr**AFYSC*Pr*IB *by In-Fusion cloning* | | |
| Insert IB PCR  Vector opening | CATATGACCACCAAAAAAGCC  AAGTCATAGCATTTACAGCAGGCCCAGCAGGCG  TAAATGCTATGACTTCACAGCTAACACCACGTCGTC  TTTGGTGGTCATATGTATATCTCCTTCTTAAAAGTTAAACAAAATTATTTGTAGAGG | p*Pr*AFYSCIB  p*Pr*Δ*L*AFYSC |
| *Assembly of* p*Tr*AFYSCIB *by In-Fusion cloning* | | |
| AFYSCIB PCR    Vector opening | GTGAGCGGATAACAATTTTGTTTAACTTTAAGAAGGAGATATACCATGGG  GTTTTATTTGATGCCTGGAGATCCTTACTCG  GGCATCAAATAAAACGAAAGGCTCAGTCG  TTGTTATCCGCTCACAATTCCACACATTATACG | p*Pr*AFYSCIB  pBbE1k-RFP |
| *Modification to produce* p*Tr*AFYSC*Tr*IB | | |
| *Trc* insertion | TGTGTGGAATTGTGAGCGGATAACAATTTGTTTAACTTTTAAGAAGGAGATATACATATGACCACC  CTCACAATTCCACACATTATACGAGCCGGATGATTAATTGTCAAATAAGTCATAGCATTTACAGCAGGCC | p*Tr*AFYSCIB |
| *Modification to produce* p*cTrΔL*AFYSCIB | | |
| Removal of pLacI^q^ | TTTTGTTTAACTTTAAGAAGGAGATATACCATGGGCAGCAGCCATCATCA  TCTCCTTCTTAAAGTTAAACAAAAATTCACCACCCTGAATTGACTCTCTTCCGGG | p*Tr*AFYSCIB |
| OEP = overlap extension PCR. | | |

| **Table S10.** Oligonucleotide sequences for the assembly of CvFAP-containing CoA-dependent DNA constructs in *E. coli*. | | |
| --- | --- | --- |
| **Stage** | **Oligonucleotide sequence 5’ to 3’** | **Template DNA** |
| *Assembly of* p*Tr*F_G462V_YIB *by In-Fusion cloning* | | |
| Vector opening  CvFAP PCR  YciA PCR  OEP | ATGCTATGACTTATTTTGTTTAACTTTTAAGAAGGAGATATACA  GGTATATCTCCTTCTTAAAGTTAAACAAAATTGTTATCCG  ATGGCCAGCGCAGTTGA  ATATGTTTCCTCCTTTATGCTGCAACGGTTGCCG  CCGTTGCAGCATAAAGGAGGAAACATATGTCTGCTAACTTTACTGATAAAAACGGTCGTCA  TTATAAGGGCTGTTCACTAATCAAGGCTAAGG  AGAAGGAGATATACCATGGCCAGCGCAGTTGA  AATAAGTCATAGCATTTATAAGGGCTGTTCACTAATCAAGGCT | p*Tr*AFYSCIB  pCvFAP_G462V_  p*Pr*oAFYSCIB  CvFAP & YciA |
| *Assembly of* p*Pr*F_G462V_YIB *by In-Fusion cloning* | | |
| Vector opening  CvFAP PCR  YciA PCR  OEP | ATGCTATGACTTATTTTGTTTAACTTTTAAGAAGGAGATATACA  GGTATATCTCCTTCTTAAAGTTAAACAAAAAAGTTAAACAAAATTATTTG  ATGGCCAGCGCAGTTGA  ATATGTTTCCTCCTTTATGCTGCAACGGTTGCCG  CCGTTGCAGCATAAAGGAGGAAACATATGTCTGCTAACTTTACTGATAAAAACGGTCGTCA  TTATAAGGGCTGTTCACTAATCAAGGCTAAGG  AGAAGGAGATATACCATGGCCAGCGCAGTTGA  AATAAGTCATAGCATTTATAAGGGCTGTTCACTAATCAAGGCT | p*Pr*AFYSCIB  pCvFAP_G462V_  p*Pr*oAFYSCIB  CvFAP & YciA |
| *Assembly of* p*Tr*CvFAP_G462V_ *by In-Fusion cloning* | | |
| Vector opening  CvFAP PCR | GGATCCAAACTCGAGTAAGGATCTCC  ATGTATATCTCCTTCTTAAAAGATCTTTTGAATTCTGAAATTGT  GAAGGAGATATACATTTATGCTGCAACGGTTGCCG  CTCGAGTTTGGATCCATGGCCAGCGCAG | pBbA1c-RFP  pCvFAP_G462V_ |
| OEP = overlap extension PCR. | | |

| **Table S11.** Oligonucleotide sequences for the assembly of KdcA-dependent DNA constructs in *E. coli*. | | |
| --- | --- | --- |
| **Stage** | **Oligonucleotide sequence 5’ to 3’** | **Template DNA** |
| *Assembly of* p*Tr*IaK*Tr*KF_G462_**_V_** *by In-Fusion cloning* | | |
| Vector opening  CvFAP PCR  ilvE PCR  αKGSDH PCR  pTrc PCR  KdcA PCR  OEP | TTCTTTATCCTCCTTCTTAAAAGATCTTTTGAATTCTGAAATTGTTATCCGCTC  GGATCCAAACTCGAGTAAGGATCTCC  CAGAACAAATAAAGGAGGATAAAGAAATGGCCAGCGCAGTTGAAG  CCTTACTCGAGTTTGGATCCTTATGCTGCAACGGTTGCCGG  TTAAGAAGGAGGATAAAGAAATGACCACCAAAAAAGCCGATTACATTTGG  TTCTTTATCCTCCTTCACTCGAGCTGATTAACCTGATCCAG  CAGCTCGAGTGAAGGAGGATAAAGAAATGGCCAATGTGACCTATACCG  GACACCATCGAATGGTGCAAAACCTTTCGCGG  CCACGGTATACATTTCTTTATCCTCCTTCTTAAAAGATCTTTTGAATTCTGAAATTGTTATCCGC  CATTATACGAGCCGGATGATTAATTGTCAATTAAACGGCCATAACGGTAACGCTTTTGG  ATCTTTTAAGAAGGAGGATAAAGAAATGTATACCGTGGGTGATTATCTGC  GGCCATTTCTTTATCCTCCTTTATTTGTTCTGTTCCGCAAACAGTTTGC  GACACCATCGAATGGTGCAAAACCTTTCGCGG  GGCCATTTCTTTATCCTCCTTTATTTGTTCTGTTCCGCAAACAGTTTGC | pBbE1k-RFP  pCvFAP_G462V_  pilvE  pαKGSDH  pBbE1k-RFP  pKdcA  pTrc & KdcA |
| *Assembly of* p*Tr*IP*Tr*KF_G462_**_V_** *by In-Fusion cloning* | | |
| Vector opening  CvFAP PCR  ilvE PCR  PadA PCR  pTrc PCR  KdcA PCR  OEP | TTCTTTATCCTCCTTCTTAAAAGATCTTTTGAATTCTGAAATTGTTATCCGCTC  GGATCCAAACTCGAGTAAGGATCTCC  CAGAACAAATAAAGGAGGATAAAGAAATGGCCAGCGCAGTTGAAG  CCTTACTCGAGTTTGGATCCTTATGCTGCAACGGTTGCCGG  TTAAGAAGGAGGATAAAGAAATGACCACCAAAAAAGCCGATTACATTTGG  TTCTTTATCCTCCTTCACTCGAGCTGATTAACCTGATCCAG  CAGCTCGAGTGAAGGAGGATAAAGAAATGACCGAACCGCATGTTGCAGT  CATTATACGAGCCGGATGATTAATTGTCAATCACTCGAGATAACGAACACAAACGCTTTTGG  CCACGGTATACATTTCTTTATCCTCCTTCTTAAAAGATCTTTTGAATTCTGAAATTGTTATCCGC  CATTATACGAGCCGGATGATTAATTGTCAATTAAACGGCCATAACGGTAACGCTTTTGG  ATCTTTTAAGAAGGAGGATAAAGAAATGTATACCGTGGGTGATTATCTGC  GGCCATTTCTTTATCCTCCTTTATTTGTTCTGTTCCGCAAACAGTTTGC  GACACCATCGAATGGTGCAAAACCTTTCGCGG  GGCCATTTCTTTATCCTCCTTTATTTGTTCTGTTCCGCAAACAGTTTGC | pBbE1k-RFP  pCvFAP_G462V_  pilvE  pPadA  pBbE1k-RFP  pKdcA  pTrc & KdcA |
| *Assembly of* p*Tr*IH*Tr*KF_G462_**_V_** *by In-Fusion cloning* | | |
| Vector opening  CvFAP PCR  ilvE PCR  HpadPCR  pTrc PCR  KdcA PCR  OEP | TTCTTTATCCTCCTTCTTAAAAGATCTTTTGAATTCTGAAATTGTTATCCGCTC  GGATCCAAACTCGAGTAAGGATCTCC  CAGAACAAATAAAGGAGGATAAAGAAATGGCCAGCGCAGTTGAAG  CCTTACTCGAGTTTGGATCCTTATGCTGCAACGGTTGCCGG  TTAAGAAGGAGGATAAAGAAATGACCACCAAAAAAGCCGATTACATTTGG  TTCTTTATCCTCCTTCACTCGAGCTGATTAACCTGATCCAG  CAGCTCGAGTGAAGGAGGATAAAGAAATGGACTTTCATCATCTGGCCTATTGG  CATTATACGAGCCGGATGATTAATTGTCAATCATGCTTCCAGGCTAATCCAAATGGTTTTCAG  CCACGGTATACATTTCTTTATCCTCCTTCTTAAAAGATCTTTTGAATTCTGAAATTGTTATCCGC  CATTATACGAGCCGGATGATTAATTGTCAATTAAACGGCCATAACGGTAACGCTTTTGG  ATCTTTTAAGAAGGAGGATAAAGAAATGTATACCGTGGGTGATTATCTGC  GGCCATTTCTTTATCCTCCTTTATTTGTTCTGTTCCGCAAACAGTTTGC  GACACCATCGAATGGTGCAAAACCTTTCGCGG  GGCCATTTCTTTATCCTCCTTTATTTGTTCTGTTCCGCAAACAGTTTGC | pBbE1k-RFP  pCvFAP_G462V_  pilvE  pHpad  pBbE1k-RFP  pKdcA  pTrc & KdcA |
| *Mutagenesis to generate* p*Tr*IaK*Tr*KF_G462_**_I_** | | |
| V462I PCR | GATCCGGATATTGTTAGCACCTATG  CAGTGCCATACCAGGAACAAAAC | p*Tr*IaK*Tr*KF_G462_**_V_** |
| *Mutagenesis to generate* p*Tr*IP*Tr*KF_G462_**_I_** | | |
| V462I PCR | GATCCGGATATTGTTAGCACCTATG  CAGTGCCATACCAGGAACAAAAC | p*Tr*IP*Tr*KF_G462_**_V_** |
| *Mutagenesis to generate* p*Tr*IH*Tr*KF_G462_**_I_** | | |
| V462I PCR | GATCCGGATATTGTTAGCACCTATG  CAGTGCCATACCAGGAACAAAAC | p*Tr*IH*Tr*KF_G462_**_V_** |
| *Assembly of* p*Tr*IHKF_G462_**_I_** *by self-In-Fusion cloning* | | |
| Vector opening | TGAAGGAGGATAAAGAAATGTATACCGTGGGTGATTATCTGCTGG  TTCTTTATCCTCCTTCATCATGCTTCCAGGCTAATCCAAATGGTTTTCAG | p*Tr*IH*Tr*KF_G462I_ |
| *Assembly of* p*Tr*IH*Tr*KF_G462_**_I_**E1a *by In-Fusion cloning* | | |
| Vector opening  Construct PCR | TTCTTTATCCTCCTTCTTAAAAGATCTTTTGAATTCTGAAATTGTTATCCGCTC  GGATCCAAACTCGAGTAAGGATCTCC  TTAAGAAGGAGGATAAAGAAATGACCACCAAAAAAGCCGATTACATTTGG  CCTTACTCGAGTTTGGATCCTTATGCTGCAACGGTTGCCGG | pBbE1a-RFP  p*Tr*IH*Tr*KF_G462_**_I_** |
| *Assembly of* p*Tr*IHKF_G462_**_I_**E1a *by In-Fusion cloning* | | |
| Vector opening  Construct PCR | TTCTTTATCCTCCTTCTTAAAAGATCTTTTGAATTCTGAAATTGTTATCCGCTC  GGATCCAAACTCGAGTAAGGATCTCC  TTAAGAAGGAGGATAAAGAAATGACCACCAAAAAAGCCGATTACATTTGG  CCTTACTCGAGTTTGGATCCTTATGCTGCAACGGTTGCCGG | pBbE1a-RFP  p*Tr*IHKF_G462_**_I_** |
| *Assembly of* p*cΔL*IHKF_G462_**_I_** *by self-In-Fusion cloning* | | |
| Vector opening | TTTCAGAATTCAAAAGATCTTTTAAGAAGGAGGATAAAGAAATGACCAC  GATCTTTTGAATTCTGAAAATTCACCACCCTGAATTGACTCTCTTCCG | p*Tr*IHKF_G462I_ |
| *Assembly of* p*cTrΔL*IHKF_G462_**_I_** *by self-In-Fusion cloning* | | |
| Vector opening | TCCGGCTCGTATAATGTTTCAGAATTCAAAAGATCTTTTAAGAAGGAGG  ATTATACGAGCCGGATGATTAATTGTCAAGACGTCGGAATTGCCAGCTG | p*Tr*IHKF_G462I_ |
| OEP = overlap extension PCR. Self In-Fusion cloning = PCR opening and elimination of target DNA, followed by re-circularisation of the plasmid by In-Fusion cloning in the absence of an insert. | | |

| **Table S12.** Oligonucleotide sequences for the assembly of KdcA-dependent DNA constructs in *Halomonas*. | | |
| --- | --- | --- |
| **Stage** | **Oligonucleotide sequence 5’ to 3’** | **Template DNA** |
| *Assembly of* pH*T7L*-RFP *by In-Fusion cloning* | | |
| Vector opening  RFP PCR | CTCGAGTCTGGTAAAGAAACCGCTGC  GGTATATCTCCTTCTTAAAGTTAAACAAACTAGTATTTCTC  AGAAGGAGATATACCATGGCGAGTAGCGAAGACGTTATC  TTTACCAGACTCGAGTTAAGCACCGGTGGAGTGACGAC | pHal2CvFAP_G462V_ [2]  pBbE1c-RFP |
| *Assembly of* pH*T7L*IHKF_G462I_ *by In-Fusion cloning* | | |
| Vector opening  Construct PCR | GGTATATCTCCTTCTTAAAGTTAAACAAACTAGTATTTCTCCTC  CGGCAACCGTTGCAGCATAATTAACCTAGGCTGCTGCCACC  AGAAGGAGATATACCATGACCACCAAAAAAGCCGATTACATTTGGTTTA  TTATGCTGCAACGGTTGCCG | pH*T7L*-RFP  p*Tr*IHKF_G462I_ |
| *Assembly of* pH*cT7LΔL*IHKF_G462I_ *by self In-Fusion cloning* | | |
| Vector opening | CCCTATAATGCCACAAATATAATTAACTCATGGGCAGG  TGTGGCATTATAGGGAGCTGTCACCGGATGTGCTTTCCG | pH*T7L*IHKF_G462I_ |
| *Assembly of* pH*cΔL*IHKF_G462I_ *by In-Fusion cloning* | | |
| Vector opening  Construct PCR | CAGCTCGAGTGAAGGAGGATAAAGAAATGGACTTTCATCATCTGGCCTATTGG  CCTTGCCTAGGCGGCCTCCTGTG  GCCGCCTAGGCAAGGGACACCATCGAATGGTGCAAAACCTTTCGCGG  TTCTTTATCCTCCTTCACTCGAGCTGATTAACCTGATCCAG | pH*T7L*IHKF_G462I_  p*cΔL*IHKF_G462I_ |
| *Assembly of* pH*c102*IHKF_G462I_ *by In-Fusion cloning* | | |
| Vector opening  Construct PCR  *c102* promoter | TCGGCTTTTTTGGTGGTCATCTAGTATTTCTCCTCTTTCTCTAGTAAAG  CGGCAACCGTTGCAGCATAATTAACCTAGGCTGCTGCCACC  ATGACCACCAAAAAAGCCGATTACATTTG  TTATGCTGCAACGGTTGCCG  TTGCGT**CCTGATCGTAGTGC**GTATAGAGTTTGAGAC*TTTACTAGAGAAAGAGGAGAAATACTAG* | pH*c102*-RFP^a^  p*Tr*IHKF_G462I_ |
| *Assembly of* pH*c69*IHKF_G462I_ *by In-Fusion cloning* | | |
| Vector opening  Construct PCR  *c69* promoter | TCGGCTTTTTTGGTGGTCATCTAGTATTTCTCCTCTTTCTCTAGTAAAG  CGGCAACCGTTGCAGCATAATTAACCTAGGCTGCTGCCACC  ATGACCACCAAAAAAGCCGATTACATTTG  TTATGCTGCAACGGTTGCCG  TTGCGT**GCTCATTGGCCAAT**GTATAGAGTTTGAGAC*TTTACTAGAGAAAGAGGAGAAATACTAG* | pH*c69*-RFP^a^  p*Tr*IHKF_G462I_ |
| *Assembly of* pH*c59*IHKF_G462I_ *by In-Fusion cloning* | | |
| Vector opening  Construct PCR  *c59* promoter | TCGGCTTTTTTGGTGGTCATCTAGTATTTCTCCTCTTTCTCTAGTAAAG  CGGCAACCGTTGCAGCATAATTAACCTAGGCTGCTGCCACC  ATGACCACCAAAAAAGCCGATTACATTTG  TTATGCTGCAACGGTTGCCG  TTGCGT**TGGCCACTGAGCA**GTATAGAGTTTGAGAC*TTTACTAGAGAAAGAGGAGAAATACTAG* | pH*c59*-RFP^a^  p*Tr*IHKF_G462I_ |
| ^a^pHal2-RFP plasmid [2] modified by the swapping of the T7-like promoter for a modified pPorin promoter. Underlined: -35 and -10 boxes; bold: variable region; *italics*: Shine Dalgarno and spacer sequence. Self In-Fusion cloning = PCR opening and elimination of target DNA, followed by re-circularisation of the plasmid by In-Fusion cloning in the absence of an insert. | | |

| **Table S13.** Oligonucleotide sequences for the chromosomal integration of the KdcA-dependent DNA constructs into *Halomonas*. | | |
| --- | --- | --- |
| **Stage** | **Oligonucleotide sequence 5’ to 3’** | **Template DNA** |
| *Assembly of* N3*T7L*IHKF_G462I_ *by In-Fusion cloning* | | |
| Vector opening  Construct PCR | GGTATATCTCCTTCTTAAAGTTAAACAAACTAGTATTTCTCCTC  CGGCAACCGTTGCAGCATAATTAACCTAGGCTGCTGCCACC  AGAAGGAGATATACCATGACCACCAAAAAAGCCGATTACATTTGGTTTA  TTATGCTGCAACGGTTGCCG | pSH-N3  p*Tr*IHKF_G462I_ |
| *Assembly of* N3*cT7LΔL*IHKF_G462I_ *by self In-Fusion cloning* | | |
| Vector opening | CCCTATAATGCCACAAATATAATTAACTCATGGGCAGG  TGTGGCATTATAGGGAGCTGTCACCGGATGTGCTTTCCG | N3*T7L*IHKF_G462I_ |
| *Assembly of* N3*cΔL*IHKF_G462I_ *by In-Fusion cloning* | | |
| Vector opening  Construct PCR | CAGCTCGAGTGAAGGAGGATAAAGAAATGGACTTTCATCATCTGGCCTATTGG  CCTTGCCTAGGCGGCCTCCTGTG  GCCGCCTAGGCAAGGGACACCATCGAATGGTGCAAAACCTTTCGCGG  TTCTTTATCCTCCTTCACTCGAGCTGATTAACCTGATCCAG | N3*T7L*IHKF_G462I_  p*cΔL*IHKF_G462I_ |
| *Assembly of* N3*c69*IHKF_G462I_ *by In-Fusion cloning* | | |
| Vector opening  Construct PCR | CGGCAACCGTTGCAGCATAATTAACCTAGGCTGCTGCCACC  GGCATCAAATAAAACGAAAGGCTCAGTCG  GTTTTATTTGATGCCTTTAATTAAAGCGGATAACAATTTCACAC  TTATGCTGCAACGGTTGCCG | pSH-N3  pH*c69*IHKF_G462I_ |
| *Assembly of* N15*T7L*IHKF_G462I_ *by In-Fusion cloning* | | |
| Vector opening  Construct PCR | GGTATATCTCCTTCTTAAAGTTAAACAAACTAGTATTTCTCCTC  CGGCAACCGTTGCAGCATAATTAACCTAGGCTGCTGCCACC  AGAAGGAGATATACCATGACCACCAAAAAAGCCGATTACATTTGGTTTA  TTATGCTGCAACGGTTGCCG | pSH-N15  p*Tr*IHKF_G462I_ |
| *Assembly of* N15*cT7LΔL*IHKF_G462I_ *by self In-Fusion cloning* | | |
| Vector opening | CCCTATAATGCCACAAATATAATTAACTCATGGGCAGG  TGTGGCATTATAGGGAGCTGTCACCGGATGTGCTTTCCG | N15*T7L*IHKF_G462I_ |
| *Assembly of* N15*cΔL*IHKF_G462I_ *by In-Fusion cloning* | | |
| Vector opening  Construct PCR | CAGCTCGAGTGAAGGAGGATAAAGAAATGGACTTTCATCATCTGGCCTATTGG  CCTTGCCTAGGCGGCCTCCTGTG  GCCGCCTAGGCAAGGGACACCATCGAATGGTGCAAAACCTTTCGCGG  TTCTTTATCCTCCTTCACTCGAGCTGATTAACCTGATCCAG | N15*T7L*IHKF_G462I_  p*cΔL*IHKF_G462I_ |
| *Assembly of* N15*c59*IHKF_G462I_ *by In-Fusion cloning* | | |
| Vector opening  Construct PCR | CGGCAACCGTTGCAGCATAATTAACCTAGGCTGCTGCCACC  GGCATCAAATAAAACGAAAGGCTCAGTCG  GTTTTATTTGATGCCTTTAATTAAAGCGGATAACAATTTCACAC  TTATGCTGCAACGGTTGCCG | pSH-N15  pH*c59*IHKF_G462I_ |
| Self In-Fusion cloning = PCR opening and elimination of target DNA, followed by re-circularisation of the plasmid by In-Fusion cloning in the absence of an insert. | | |

**References**

1. Bartolomeo MP, Maisano F. Validation of a reversed-phase HPLC method for quantitative amino acid analysis. J Biomol Tech. 2006;17:131-7.

2. Amer M, Wojcik EZ, Sun C, Hoeven R, Hughes JMX, Faulkner M, Yunus IS, Tait S, Johannissen LO, Hardman SJO *et al*. Low carbon strategies for sustainable bio-alkane gas production and renewable energy. Energy Environ Sci. 2020; doi:10.1039/d0ee00095g.

3. Sezonov G, Joseleau-Petit D, D'Ari R. *Escherichia coli* physiology in Luria-Bertani broth. J Bacteriol. 2007;189:8746-9.

4. Menon N, Pásztor A, Menon BRK, Kallio P, Fisher K, Akhtar MK, Leys D, Jones PR, Scrutton NS. A microbial platform for renewable propane synthesis based on a fermentative butanol pathway. Biotechnol Biofuels. 2015;8:61.

5. Hoeven R, Hughes JMX, Amer M, Wojcik EZ, Tait S, Faulkner M, Yunus IS, Hardman SJO, Johannissen LO, Chen G-Q *et al*. Distributed biomanufacturing of liquefied petroleum gas. Submitted. 2019.

6. Qin Q, Ling C, Zhao Y, Yang T, Yin J, Guo Y, Chen GQ. CRISPR/Cas9 editing genome of extremophile *Halomonas* spp. Metab Eng. 2018;47:219-29.

7. Sabri S, Steen JA, Bongers M, Nielsen LK, Vickers CE. Knock-in/Knock-out (KIKO) vectors for rapid integration of large DNA sequences, including whole metabolic pathways, onto the *Escherichia coli* chromosome at well-characterised loci. Microb Cell Fact. 2013;12:60.

8. Davis JH, Rubin AJ, Sauer RT. Design, construction and characterization of a set of insulated bacterial promoters. Nucleic Acids Res. 2011;39:1131-41.

9. Lee TS, Krupa RA, Zhang F, Hajimorad M, Holtz WJ, Prasad N, Lee SK, Keasling JD. BglBrick vectors and datasheets: A synthetic biology platform for gene expression. J Biol Eng. 2011;5:15-7.

10. Zhao H, Zhang HM, Chen X, Li T, Wu Q, Ouyang Q, Chen G-Q. Novel T7-like expression systems used for *Halomonas*. Metab Eng. 2017;39:128-40.
